# A Scoping Review of Empirical Research Relating to Quality and Effectiveness of Research Ethics Review

**DOI:** 10.1371/journal.pone.0133639

**Published:** 2015-07-30

**Authors:** Stuart G. Nicholls, Tavis P. Hayes, Jamie C. Brehaut, Michael McDonald, Charles Weijer, Raphael Saginur, Dean Fergusson

**Affiliations:** 1 School of Epidemiology, Public health and Preventive Medicine, University of Ottawa, Ottawa, Ontario, Canada; 2 Ottawa Hospital Research Institute, Clinical Epidemiology Program, Ottawa, Ontario, Canada; 3 The W. Maurice Young Centre for Applied Ethics, The University of British Columbia, Vancouver, British Columbia, Canada; 4 Rotman Institute of Philosophy, Western University, London, Ontario, Canada; Cardiff University, UNITED KINGDOM

## Abstract

**Background:**

To date there is no established consensus of assessment criteria for evaluating research ethics review.

**Methods:**

We conducted a scoping review of empirical research assessing ethics review processes in order to identify common elements assessed, research foci, and research gaps to aid in the development of assessment criteria. Electronic searches of Ovid Medline, PsychInfo, and the Cochrane DSR, ACP Journal Club, DARE, CCTR, CMR, HTA, and NHSEED, were conducted. After de-duplication, 4234 titles and abstracts were reviewed. Altogether 4036 articles were excluded following screening of titles, abstracts and full text. A total of 198 articles included for final data extraction.

**Results:**

Few studies originated from outside North America and Europe. No study reported using an underlying theory or framework of quality/effectiveness to guide study design or analyses. We did not identify any studies that had involved a controlled trial - randomised or otherwise – of ethics review procedures or processes. Studies varied substantially with respect to outcomes assessed, although tended to focus on structure and timeliness of ethics review.

**Discussion:**

Our findings indicate a lack of consensus on appropriate assessment criteria, exemplified by the varied study outcomes identified, but also a fragmented body of research. To date research has been largely quantitative, with little attention given to stakeholder experiences, and is largely cross sectional. A lack of longitudinal research to date precludes analyses of change or assessment of quality improvement in ethics review.

## Background

Research ethics review was developed by a post-WWII society to ensure that human subjects were protected from unethical research. Today ethical review is legally mandated prior to the conduct of most human subjects research [1].

While few would disagree with the general need for ethics review, existing review processes are often criticized [2]; common complaints include the amount of paperwork required [3], inconsistency of decisions between review boards, and suggestions that ethics review systems may not be equipped to properly review specific types of research [4–8]. In response to these criticisms, efforts have been made to develop standards of ethics review, and several jurisdictions have implemented accreditation processes to ensure that committees meet requirements, such as those imposed by the US Federal Policy for the Protection of Human Subjects (the ‘Common Rule’)[9]. However, these largely procedural standards may not necessarily reflect the goals of human subject protection that review processes were established to safeguard. To date, there is no established consensus regarding assessment criteria for evaluating research ethics review [10].

Abstract goals and evaluative frameworks have been described [11], but there remain a lack of operational definitions and consensus regarding criteria against which to perform assessments. Indeed, while there has been much discussion of the need to develop metrics or quality indicators, there has been little progress in terms of identifying and testing meaningful indicators. Despite a recent systematic review to determine what is known about how well IRBs function [12], several existing areas of study were excluded. Indeed, despite the conclusion that there is a need to clarify expectations regarding ethics review processes, and that data on the risks that research participants experience would be helpful in this regard, the authors explicitly excluded stakeholder opinions of IRB performance. Moreover, the review did not explore in detail the different methodological approaches, stakeholders involved, or theories motivating the research.

In order to progress the literature towards evidence-based assessment of ethics review processes, there is a need to examine not just procedural aspects of ethics review, but also a broader range of perspectives and descriptive accounts as well as a range of methodological approaches. In the present review we address this need through an inclusive search of the international literature, and specifically include studies targeting investigator, participant, and research board/committee perspectives with attention given to methodological approach.

## Aim

To conduct a scoping review of the relevant literature regarding the evaluation of research ethics review, and to summarize the available evidence in terms of:

1. Applied theoretical frameworks relevant to evaluating research ethics review;
2. Research approaches that have been used to evaluate research ethics review;
3. Subjects of analysis within existing research to evaluate research ethics review; and
4. Research outcomes that have been used to evaluate research ethics review;

## Methods

Our choice to conduct a scoping review was necessitated by the disparate body of literature regarding ethics review practices. Scoping reviews are useful to summarize and describe data from a wide range of fields which cross disciplinary and methodological lines [13]. This can include quantitative, qualitative, and review work. In keeping with the aim of scoping reviews, our approach was also informed by a desire to examine the extent, range and nature of research activity so as to provide an initial assessment of the state of the literature and identify research gaps. As per recommended practice [13, 14] we used a five step framework. The five stage process employed was:

1. Identifying the research question;
2. Identifying relevant studies;
3. Study selection;
4. Charting the data;
5. Collating, summarizing and reporting the results.

### Identifying the research question

Our main question for the scoping review was: What empirical research exists that addresses the evaluation of research ethics review?

### Identifying Relevant Studies

Studies were identified through an electronic search of published literature, together with citation tracking and hand searching. Electronic searches of Ovid Medline, PsychInfo, and the Cochrane DSR, ACP Journal Club, DARE, CCTR, CMR, HTA, and NHSEED, were conducted. Terms relating to research ethics boards, quality, effectiveness and evaluation were combined with terms relating to research approaches (See S1 File). The search strategy was developed through discussion with experts in the field of research ethics review, a research librarian, a previously published systematic review [12], and a narrative review of the literature. The search strategy included both Meta Subject Heading (MeSH) terms and text words as several articles identified by the narrative review did not have MeSH terms associated with them.

### Study Selection

Eligibility criteria were based on the goals of our research question. While there has been much debate with respect to potential indicators of quality in research ethics review, our goal was to advance the empirical assessment of ethics review. As the motivation for the study was to move forward the research agenda on quality assessment in a meaningful way we limited our search to include only manuscripts that had attempted to develop metrics, or evaluate empirically, research ethics review processes or procedures. Studies were therefore excluded if they did not involve empirical research; did not have research ethics review (as opposed to clinical ethics review) as a core element of study; or didn’t relate to humans (e.g. studies of animal research ethics). Articles were not limited by date, allowing the assessment of publication trends. Only English language studies were included.

The electronic search was conducted in June 2013 and updated in March 2014. All titles and abstracts were screened by two reviewers (TH, SN). Following the initial screen, the bibliographies of all retained articles were hand searched to identify additional studies. All articles were imported into Reference Manager 12 for curation. Articles were rejected on an initial screen of titles and abstracts only if the reviewers could determine that the articles did not meet the inclusion criteria. Where abstracts were not available, or where a definitive assessment could not be made, the full text of the article was retrieved. The same two authors reviewed the full texts to make a final determination of inclusion or exclusion. Any disagreements were resolved by discussion. Data extraction was conducted by one reviewer (TH), with a second reviewer (SN) screening a sample (n = 45) for comparison. Each reviewer independently extracted information from the full text manuscript and then results were compared. Differences that were qualitatively different (i.e. there had been different elements extracted) were resolved through discussion as were differences in coding applied to the data.

### Charting the data

A data extraction form and process was developed based on the study aim of creating a descriptive account of the research landscape, as opposed to integrated analyses. The content of the form was developed by discussion within the team. Data extracted included: article characteristics (title, author(s), source, date of publication); description of research (type of participants, study design, data collection methods, research question, study size, dates to which data relate, region); and study outcomes.

### Collating, summarizing and reporting results

Data were summarized descriptively. Qualitative data, such as individual outcomes from studies or descriptions of approaches, were collated thematically using a process of qualitative description. This is a low-inference approach to coding qualitative data in which the goal is a descriptive account of the content, as opposed to overarching concepts or abstract frameworks[15]. Themes were applied using the constant comparison method in which existing instances are revisited in light of new data. [16] Descriptive statistics were used to explore the quantitative data within the manuscripts. The data extracted are listed in Table 1.

**Table 1:** Extracted information from retrieved articles.

Article Characteristics	Description of Research	Study Findings and Conclusions
Title	Type of Participants	Names of outcomes
Author(s)	Study Design	Results/Findings
Source	Research Questions	Theoretical framework/theory cited? If yes: definition
Date of Publication	Study Size	Authors’ Conclusions
	Dates to which data relate	
	Region	
	Definition of quality	
	Definition of effectiveness		

## Results

**Fig 1:**
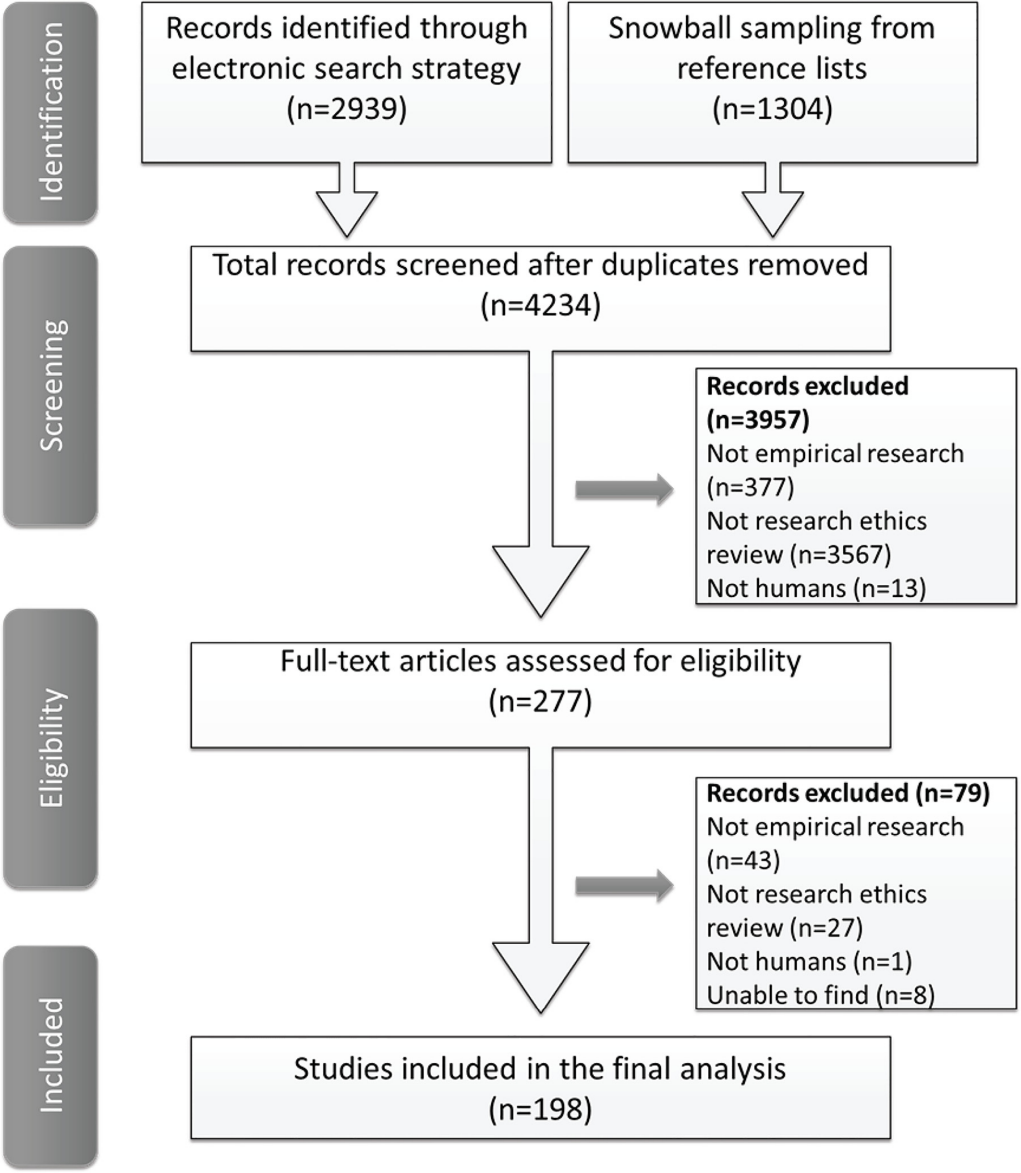
Flowchart of screening process

The electronic search resulted in 2939 citations for review. Review of bibliographies for initially retained papers yielded a further 1304 articles. After de-duplication a total of n = 4234 titles and abstracts were reviewed. Screening by both reviewers achieved 94% concordance. Altogether 4036 articles were excluded following screening of titles, abstracts and full text. The main reasons for exclusion were: not research ethics review (n = 3594), not empirical research (n = 420), not human (n = 14). In addition we were unable to locate the full text of 18 articles. Consequently, a total of 198 articles were included for final data extraction (see Fig 1).

### Study descriptors

Publication dates of identified studies ranged from 1979 to 2014. From 1979 through to the 1990s the number of studies identified number one to two per year. There was an increase in the number of articles per year starting in the early 2000s (from n = 6 in 2000 to n = 14 in 2005) with a peak in the latter part of that decade (n = 19 in 2008). Several studies did not include dates to which their data relate, precluding assessment of this. Most studies originated from North America (n = 102) or Europe (n = 62). There were relatively few authors publishing multiple articles.

### Theoretical frameworks

No study reported using an underlying theory or framework of IRB quality/effectiveness to guide study design or analyses. Several studies did, however, use theories, such as grounded theory, to analyze data [17–20].

While a number of studies (n = 16) discussed quality or effectiveness of IRB decisions [7, 12, 21–34], none provided explicit operational definitions. In developing their self-assessment tool, Sleem et al., note that “there are no gold standards for determining effectiveness nor are there standards that can actually measure how well human participants are being protected by the use of standards”, instead opting to use ‘surrogate’ metrics that they considered foundations for effectiveness and protection [30]. These surrogate metrics included: availability of policies (e.g. to deal with conflicts of interest), structural elements (such as membership composition), processes (for example, clear processes for the submission of protocols), performance measures (such as whether certain criteria were considered within the protocol review), as well as cost-related information. While the structural organization of review (for example, policies, structural elements, performance measures) is not itself a theory it does provide a framework of aspects of IRB review quality. The development of such metrics, in the absence of explicit operational definitions, was representative of many studies identified by the review.

Two studies did describe a general foundation in Procedural- and Interactional-Justice through the use of the Institutional Review Board-Researcher Assessment Tool (IRB-RAT) [35, 36]. *Procedural justice* relates to fairness of process. A fair IRB, it is argued by the authors, might display characteristics that are associated with procedural justice, such as: consistency, lack of bias, accuracy, procedures for correcting errors, representativeness, and adherence to basic ethical standards. *Interactional justice*, on the other hand, relates the behavioral aspects of the decision process. In this respect, the authors of the IRB-RAT argue for the inclusion of this aspect to evaluate the way in which people who receive decisions are treated. In essence, it is an evaluation of communication through assessment of *interpersonal sensitivity*–the degree of politeness or respect–and the substantive *justification*, that is the degree of explanation provided.

### Research approaches

**Fig 2:**
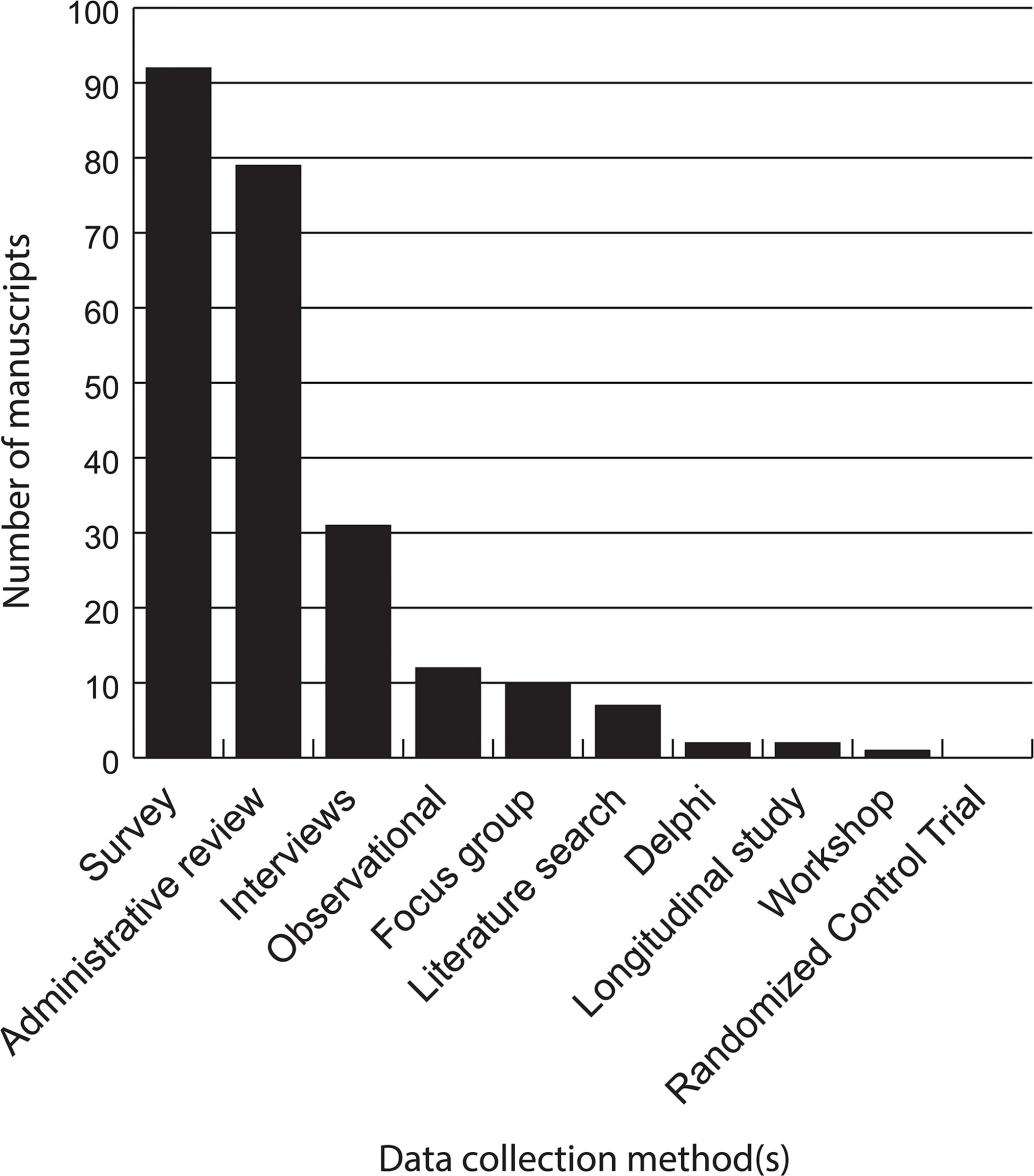
Data collection methods of analysed manuscripts.

We did not identify any studies that had involved a controlled trial—randomised or otherwise–of ethics review procedures or processes. The two most common methods of data collection were surveys, with 92/198 (46%) manuscripts reporting results from survey research, and review of administrative data, with 79 (40%) papers (Fig 2 and S1 Table, for further details). Survey respondents varied, with manuscripts reporting on surveys with several populations. Of the 92 manuscripts reporting survey research, 63 included surveys of ethics committee/board members (69%), 28 included surveys of researchers (31%), and 4 included surveys of research participants (4%). Surveys also often focused on structural aspects of ethics review, with 52 (57%) manuscripts exploring structural or procedural aspects, 43 (47%) elements of membership, and 27 (29%) variation, while 39 (42%) explored ethics committee/board member views. Eighteen manuscripts included researcher views (20%) and 3/92 (3%) papers using surveys included participant views.

Thirty one papers (16%) reported data collected through interviews. Of these, 4 manuscripts reported interviews with research participants (13%), 8 included researchers (26%) and 24 (77%) were with ethics committee/board members. A handful of studies reported results from other qualitative approaches such as participant observation (n = 12; 6%) or focus groups (n = 10, 5%).

Seven papers indicated that a literature review had been undertaken: however, in only two instances were detailed search strategies and summaries of the identified literature provided [12, 29].We identified two examples of Delphi processes [26, 37] and only two studies of longitudinal data [38, 39]. Of the two longitudinal studies, Denham et al., reviewed the outcomes of studies reviewed and approved by a single research ethics committee in the UK over the period 1970 to 1978. Based on follow up they found that 43% of projects approved had been completed, 20% had been abandoned, 3% had been suspended and 26% were ongoing [38]. Allen and Waters reviewed the data on number of projects submitted, the types of study, and the numbers approved and requiring modification–including details on the types of modifications or conditions imposed by the ethics committee [39].One manuscript presented a summary of a workshop [40].

### Research subject

The research subject referred to what, or who, was the subject of analysis (S1 Table). A total of 147/198 papers reported data where the assessment of administrative processes was the subject of assessment (74%), while 103 (52%) reported the views of IRB members. A total of 45 manuscripts (23%) related to analyses of review board composition, and 37 (19%) explored the views of researchers. A handful of papers included alternative subjects of study. Eight manuscripts explored the views of non-research healthcare workers who may be affected by research [37, 41–47], and only seven papers (4%) identified by the search involved research participants as the subject of study [48–54]. We identified only one study that explored the views of the research sponsors[55].

### Outcomes of assessment/thematic analyses

Table 2 describes the themes we identified: Membership; Time; Cost; Variation; Satisfaction; Policy Adherence; Working Hours; Outcome; Training; Knowledge; Structures and Procedures; Number of Protocols; Committee/board Member Views; Researcher Views; Participant Views; Committee/board Decision Making; Post Approval Monitoring; Number of Committee/boards in Region; and Views of Healthcare Professionals (HCPs) (see Table 2 for examples of individual outcomes included within the thematic groupings). Studies often assessed multiple outcomes.

**Table 2:** Examples of individual study outcomes according to thematic groupings.

Thematic grouping	Examples
Membership	**Borovecki et al (2005):**1) IRB membership information: age, sex, occupation 2) Number of members in the committee
	**Catania et al (2008):** 1) The composition of each IRB committee administered by their office: total members per committee, number of non-institutional members, number of non-institutional members without a science background
Time	**Ahmed et al (1996):** 1) Time taken (days) to obtain ethical approval
	**Al-Shahi et al (1999):** Delay from application to- 1) Calling an LREC meeting 2)Initial LREC decision 3) Final LREC approval
Cost	**Byrne et al (2006):** Number of units of various resources that were used at a given IRB. 1)Travel 2)Supply and equipment purchases 3) Space used
Cost	**Chakladar et al (2011):** 1) Number of sheets of A4 paper distributed to committee members and used during requested amendments or resubmissions. 2) Paper use during IRB process. 3) Paper use during study conduct
Variation	**Angell et al (2006):** 1) Patterns of agreement in decisions, descriptively and using the kappa statistic.
	**Fitzgerald et al (2006) (62):** 1) Comparison between centralized and decentralized systems: administrative and the review process
Satisfaction	**Mosconi et al (2006):**1) Average level of satisfaction on the interactions with the REC for each of the following aspects: bureaucratic and secretarial, ethical, scientific and methodological, education aspects and training activities
Policy Adherence	**Abbott et al (2011)** 1) Process studies examining the extent to which federal regulations are implemented by the IRB
	**Ateudjieu et al (2010)** 1) Difficulties in applying regulations
Working Hours	**Ah-See et al (1998):** 1) Frequency of meetings
	**Kirigia et al (2005):** 1) Frequency of scheduled meetings 2) Number of times the committee actually met last year
Outcome	**Czarkowski et al (2009):** 1) Number of negative assessments given
	**Russ et al (2009):** 1) Frequency of formal and content-related objections in the decisions of coordinating ethics committees after first application
Training	**Ateudjieu et al (2010):** 1) Training on research ethics evaluation. 2) Types of Training. 3) Training Content. 4) Perceived importance of targeted groups for training. 5) Training objectives
Knowledge	**Banos et al (2010):** 1) Degree of improvement in the knowledge of those attending seminars
	**Borovecki et al (2006):** 1) Self assessment of the knowledge of each respondent in the field of biomedical ethics. 2) Participants’ knowledge on the field of biomedical ethics, bioethics issues
Structures and procedures	**Foster et al (1998):** 1) Policies regarding multi-centre research
	**Jones et al (1996):** 1) Policies concerning scientific misconduct
Number of Protocols	**Boyce (2002)** 1) Number of new and continuing applications discussed at each meeting
	**Catania et al (2008)** 1) Types and volume of protocols received in the past year. 2) Total number of protocols [new and prior] 3) Number of new [all types] and of new full-committee review protocols
IRB Member Views	**Abou-Zeid et al (2009)** 1) Self-rated capacity to perform committee activities
	**Allen et al (1983):** 1) Present and retired IRB member general attitudes towards ethical committees and their functions
Researcher Views	**Douglass et al (1998):** 1) Researcher experiences of the ethics review process
	**Kallgren et al (1996):** 1) Student researcher reactions to going through the IRB process
Participant views	**Berry (1997):** 1) Did the patients know that they were research subjects? 2) Had they been given enough information and enough time to give valid consent? 3) Had they been told what to do if there was a problem?
	**Karunaratne et al (2006):**1) Were there any parts which you found difficult to understand? 1) Which activities do you think ethics committees are involved in?
IRB Decision Making	**Boyce (2002):** 1) Reasons for condition approval/deferral
	**Czarkowski et al (2009):** 1) Basis on which decisions concerning research projects were made. 2) Basis for reviewing applications
Post Approval Monitoring	**Arda (2000):** 1) Methods used to monitor the progress of projects
	**Gibson et al (2008)** 1) Assessment of need for ongoing monitoring of registry by REB 2) Types of information that would need to be reported
Number of RECs in Region	**Vulcano (2012)** 1) Assessment of the number IRBs using a database
Views of HCPs	**Allen et al (1983):** 1) Doctors who have never been members of an ethical committee views towards ethical committees and their functions

**Fig 3:**
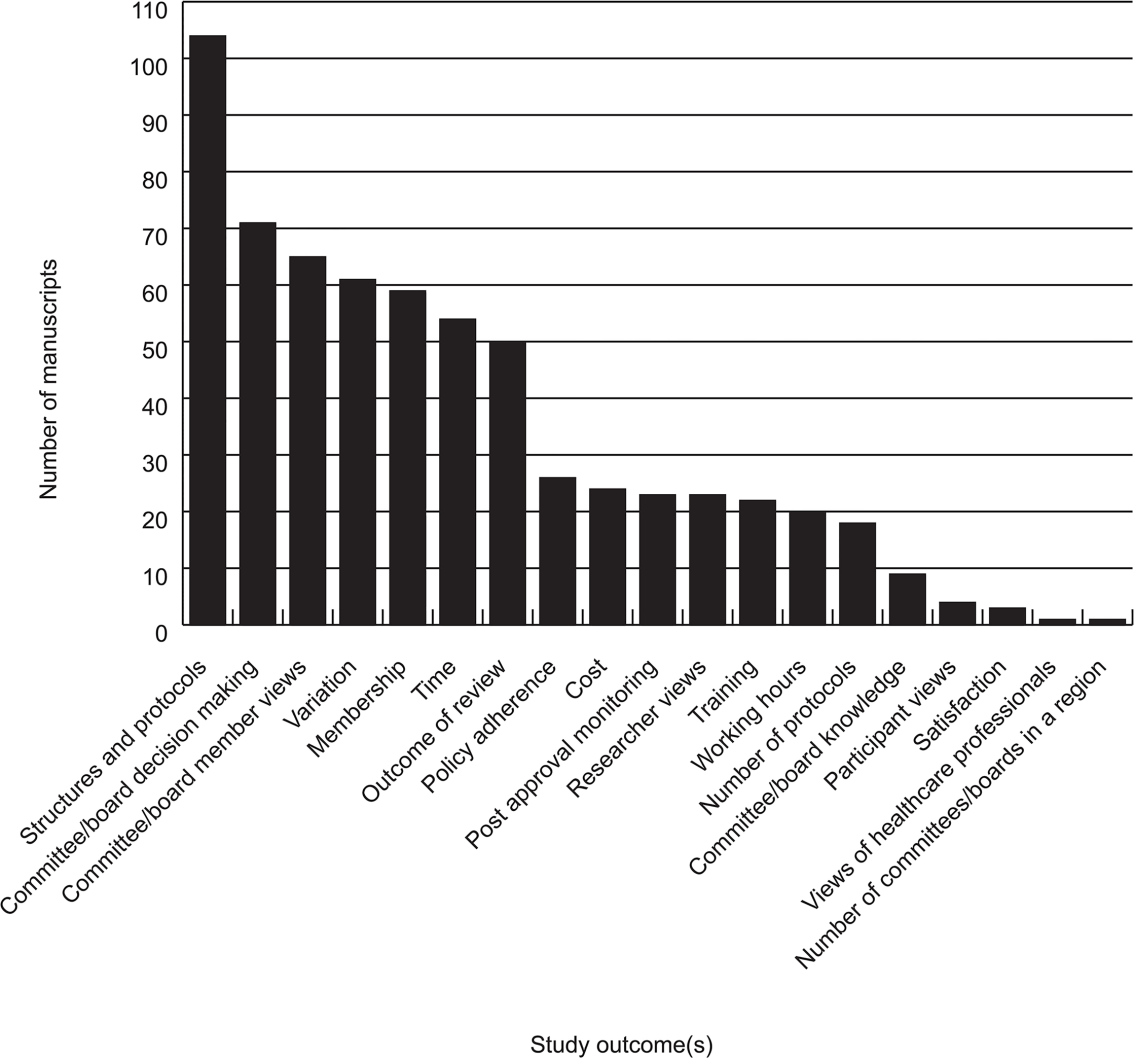
Instances of outcomes present in analysed manuscripts.

As Fig 3 shows most outcomes were situated within the cluster relating to ethics committee/board processes and outcomes (see also S1 Table). The largest number of manuscripts assessed structures and protocols of review committees or boards (n = 104, 53%). For example, Foster et al. reviewed annual reports of UK Local Research Ethics Committees (LRECs) and sought to determine their size, gender composition, and fees charged for review [56]. Other outcomes in this more common grouping were: Committee decision making (n = 71, 36%), committee/board member views (n = 65, 33%), variation between review committees/boards (n = 61, 31%), ethics committee/board membership (n = 59, 30%); time taken for review (n = 54, 28%), outcome of review (n = 50, 25%).

Within the second cluster of outcomes–which tended to represent assessments of functional aspects of committee/board approval and monitoring—the most popular outcome was the comparison of ethics review performance against existing standards or legislation, such as the Common Rule (n = 26, 13%). Of those assessing performance against existing standards, several studies reported that different IRBs varied in their interpretation and application of the same guidelines [12, 56–59]. Some authors noted that certain criteria–such as informed consent–received much greater consideration than others, such as risk minimization or data monitoring requirements [58]. Others report variation in the requirements of ethics applications, even within the same jurisdiction [59]. Other outcomes within this cluster were costs (n = 24, 12%), researcher views (n = 23, 12%), post approval monitoring (n = 23, 12%), training undertaken by review board members (n = 22, 11%), working hours (n = 20, 10%), and number of protocols reviewed (n = 18, 9%).

Least studied were outcomes relating to human subjects protections, and the conduct of others involved in the research ethics enterprise. Notably, the views of healthcare professionals not directly involved in research and research participants were rarely studied.

Nine studies identified assessed ethics committee/board member knowledge. As above, multiple approaches were often employed, with seven studies using surveys to explore knowledge, three focus groups, one study using an observational design, and another conducting interviews. These studies ranged with respect to the areas of knowledge being evaluated and how this was assessed. Allen, for example, explored IRB member knowledge of processes and procedures for reviewing genetics protocols [60] while others explored committee/board member knowledge of methodology [42] and ethical principles [42, 61–63] and procedures [55, 63, 64].

We identified four studies (2%) that specifically explored the views of research participants, and one that assessed the views of healthcare professionals not directly involved in research [41]. Studies of participant views ranged in focus, from evaluating IRB consent decisions by exploring participant experiences and understanding of the research in which they were involved [48, 50, 54] to surveying research participants regarding their views as to the roles and purposes of ethics committees [51].

### Existing tools

A number of tools were identified that could potentially provide standardized assessments of ethics boards/committees (S2 Table). These include: the IRB-RAT [35, 36], the Training and Resources in Research Ethics Evaluation (TRREE)[65], the Research Ethics Committee (REC) Quality Assurance Self-Assessment Tool[30], an assessment tool developed by Tsan et al., for evaluating research protection programs in the Department of Veterans Affairs [32, 33], and a draft evaluation instrument for use in convened NIH IRB meetings [63].

However, there has been little–if any–validation of these tools. Only one tool–the IRB-RAT–has been used in a replication study, although Tsan et al., have applied their tool at several time points to evaluate the same population [32, 33]. While the NIH instrument is reported as something that will be used to evaluate four of the NIH’s 14 IRBs, no follow up reports were identified by our review.

## Discussion

While research ethics review is a cornerstone of ethical research practice, there are no gold standards against which to evaluate research ethics review processes. This lack of standards stems, at least in part, from the lack of consensus regarding assessment criteria, but may also indicate a lack of emphasis on the evaluation of ethics review processes.

The findings of our scoping review indicate that until the turn of the 21^st^ Century there has been little in the way of published research on the subject of assessment of research ethics review. What published research there has been has varied in terms of methodological approaches, subjects of assessment, and the outcomes evaluated. Most research has been conducted into procedural aspects of research ethics review such as committee composition, variation in review outcomes or time to approval, and that the majority of research has been conducted using quantitative approaches such as surveys or administrative review of quantitative data. The majority of research that was identified in this review has been conducted in North America and Europe.

### Research approaches

The majority of studies retained in our review were quantitative in nature. As a result there has tended to be a focus on descriptive research; studies have documented how committees are composed, and the number of studies reviewed, or the amount of variation between committees reviewing the same protocol. There is much less explanatory research: why do committees make the decisions they do? How do the dynamics of committees play into decisions? Qualitative studies that include ethnographic methods could help to elucidate decision making models or objects of concern that are not easily or readily accessible through structured quantitative approaches.

A second notable gap in the existing literature is the lack of long-term–or longitudinal–assessment. The lack of longitudinal research is problematic if a goal is to protect human subjects or derive a net benefit for clinical research: as the study of de Jong et al., indicates, research outcomes, adverse events, or publications may not be immediately accessible and only through longitudinal studies would these outcomes be amenable to evaluation. Indeed, their finding that studies that had more correspondence with an ethics committee were less likely to achieve publication [66] is something that should motivate a greater degree of research into post approval monitoring.

The lack of longitudinal research may be symptomatic of the lack of a coherent research agenda with respect to developing evaluation frameworks or tools against which to assess research ethics review processes. Moreover, there may be barriers to the conduct of such research. A study by McKenzie et al., that sought to conduct long term follow up of studies receiving ethical approval itself faced difficulties in obtaining ethical approval on the grounds that the researchers were not obtaining informed consent from the trialists to view their ethics application [67].There is a need for leadership in this area, but also greater collaboration. Important questions need to be asked of researchers, administrators and funders. Funding will be central, but will also generate questions of responsibility and management: given the vagaries of short term contract research and associated funding, should the collection of information on ethics review processes be centrally resourced and conducted by ethics review committees themselves? Does this need to be done by an independent oversight body such as the Association for the Accreditation of Human Research Protection Programs (AAHRP), and if so how should this be managed and reported? These questions cannot be addressed in isolation, and need all relevant stakeholders to be at the table.

### Research subjects

Our results indicate that there has been limited research with key stakeholders beyond the membership of ethics committees/boards and the researchers that interact with them; the views of research participants have been largely missing from existing research. If a goal is to develop evaluation tools to assess research ethics review processes against their remit of protecting human subjects, then further research is warranted with those individuals who are subject to research. Indeed, current research is lacking several stakeholders who may be considered relevant to the debate. McDonald et al., have argued that research ethics review is but one part of the research ethics lifecycle, and that there are a broader range of perspectives that need to be considered beyond the researcher-ethics committee/board dyad [68]. We found little research with healthcare professionals outside the research context, and only one study that included the views of research sponsors. Identifying and including all relevant stakeholders in the review process; be they researchers, IRB members, policy-makers, legislators, research funders, institutional-sponsors, or research participants, will be key to identifying shared goals of research ethics review that are appropriate for, and amenable to, assessment. As such, we suggest that more research is needed that includes additional stakeholders beyond the IRB-researcher dyad.

### Research outcomes

Given that research ethics review has been established to minimize harms to research participants, and that existing guidelines, regulations and research indicate that the protection of human subjects is a continued goal, we found a paucity of research exploring the experiences of research participants.Greater involvement of participants (and the public) may provide greater support for the decisions made, and could potentially lead to increased trust in the decision-makers and decision-making process as well as improved decisions [69]. Moreover, exploring participants’ experiences may identify factors that contribute to potential negative effects, and facilitate modifications to the review process that may mitigate future repetition.

While calls for the development of metrics for measuring the quality of ethics review appear to have been heeded to the extent that some instruments were identified within the review, there has, to date, been little evaluation of these tools. Existing instruments reflect a fragmented research program in which individual researchers have developed custom data collection tools. This has not only limited assessments of reliability or validity, but has led to competing and contrasting data collection tools being developed.

Tools developed in other areas relating to core ethical principles could be useful for the evaluation of ethics review processes and should be considered for evaluation. In a recent review of measurement instruments in clinical and research ethics, Redman identified 10 validated instruments measuring ethical constructs [70].This included two measures of informed consent; the Multi-Dimensional Measure of Informed Choice [71], and the Quality of Informed Consent [72] instruments, but only one instrument that directly related to research. This tool, the Reactions to Research Participation Questionnaires for Children and for Parents (RRPQ-C and RRPQ-P), was developed to evaluate experiences of participating in research, as opposed to incorporating this within a framework for the evaluation of research ethics review [73]. Using tools such as this within a framework to evaluate research ethics review processes could allow for consistent metrics of assessment while specifically addressing the important goals of human subject protections. Moreover, the focus of measures such as this would clearly address the present research gap on participant experiences. However, further development and evaluation is needed to evaluate if such a tool is appropriate, together with consideration of whether this should be a researcher driven evaluation, or something undertaken by review boards themselves.

### Limitations

Our results must be interpreted within the context of the limitations of the study. Firstly, our sampling frame was limited to a specific number of databases. As such, some articles, such as articles from social science databases or grey literature, may be missing based on the limits and boundaries of the included databases. A second caveat is the specificity of the search strategy itself: while steps were taken to ensure that key articles were included, the sensitivity of the search strategy was limited in order to generate a manageable number of articles. However, our review may have been overly-calibrated toward identified key articles. We attempted to mitigate these limitations through reviewing the reference lists of articles, which was not limited by the original databases or the terms within the search strategy. The substantial number of articles achieved through this process indicates the utility of this approach in a heterogeneous area such as the evaluation of research ethics review. Finally, our search strategy was limited to English language publications. This may have biased our results towards countries where this is the predominant language of publication and may account, in part, for the larger number of articles retrieved from certain countries or geographic regions.

## Conclusion

There is a continued call for, and interest in, the development of quality indicators for research ethics review. Our review indicates a lack of consensus on appropriate assessment criteria, exemplified by the varied study outcomes identified, but also a fragmented body of research. To date research has been largely quantitative, with little attention given to stakeholder experiences, and cross sectional. On the basis of our review we make the following recommendations for future research developments:

1. Assessment of long-term outcomes following research ethics review to identify variation within and between ethics review committees and to allow time for the identification of potential trends.
2. Engagement with a broader range of stakeholders, including research participants, in order to avoid viewing research ethics solely as ethics review, as opposed to a broader research ethics lifecycle [74].
3. The development of theoretical foundations upon which to base empirical investigations of research ethics review
4. The creation of review strategies and structures that facilitate the systematic search of the diverse literature around the evaluation of research ethics review including high quality databases of peer-reviewed publications across the range of disciplines and a common interface and search language.

## Supporting Information

S1 File Search Strategy. (DOC)

S1 Table Articles retrieved. (DOC)

S2 Table Identified measures or tools for evaluating research ethics review. (DOC)
