# Supplementary material for: A Scoping Review of Empirical Research Relating to Quality and Effectiveness of Research Ethics Review
Source: PLoS One. 2015 Jul 30;10(7):e0133639. doi: 10.1371/journal.pone.0133639 (PMC4520456; doi:10.1371/journal.pone.0133639)
Supplement: S1 File — (DOC) [file pone.0133639.s001.doc]

S1 File: Search Strategy

(exp Ethics Committees/ or (ethic* adj3 committee*).tw. or (ethic* adj3 review*).tw. or "institutional review board".tw. or IRB.tw.) and (exp Questionnaires/ or quality.tw. or desirable.tw.)
