# Supplementary material for: A Scoping Review of Empirical Research Relating to Quality and Effectiveness of Research Ethics Review
Source: PLoS One. 2015 Jul 30;10(7):e0133639. doi: 10.1371/journal.pone.0133639 (PMC4520456; doi:10.1371/journal.pone.0133639)
Supplement: S1 Table — (DOC) [file pone.0133639.s002.doc]

S1 Table : Articles retrieved

| **Author** | **Location** | **Research Subject** | **Data Collection** | **Outcomes** |
| --- | --- | --- | --- | --- |
| **Abbott et al(1)** | USA | Review Process | Systematic Review | Structures and procedures, Cost, Membership, Policy Adherence, Variation, IRB Decision Making, Outcome |
| **Abou-Zeid et al(2)** | Asia, Europe | IRB Members, Composition, Review Process | Survey | Structures and procedures, Membership, IRB Member Views, Training, Policy Adherence, Variation |
| **Ahmed et al(3)** | Europe | IRB Members, Review Process | Administrative Review | Outcome, Structures and procedures, Time |
| **Ah-See et al(4)** | Europe | Review Process | Administrative Review | Structures and procedures, Policy Adherence, Working Hours, Time, IRB Decision Making |
| **Akabayashi et al(5)** | Asia | IRB Members, Composition, Review Process | Survey | Membership, Structures and procedures, IRB Decision Making, Variation, IRB Member Views |
| **Allen(6)** | USA | IRB Members, Review Process | Survey | IRB Member Views, Training, Policy Adherence, Knowledge, Structures and procedures, Time, Satisfaction |
| **Allen et al(7)** | Europe | Composition, Review Process | Administrative Review, Longitudinal Study | Membership, Number of Protocols, IRB Decision Making, IRB Member Views, Outcome |
| **Allen et al(8)** | Europe | IRB Members, Health Care Workers | Survey | IRB Member Views, HCP Views |
| **Al-Shahi et al(9)** | Europe | Review Process | Observational | Time, Cost |
| **Angell et al(10)** | Europe | IRB Members, Review Process | Administrative Review | Variation, IRB Decision Making |
| **Angell et al(11)** | Europe | Review Process | Administrative Review | Outcome, IRB Decision Making, Variation |
| **Arda(12)** | Asia | IRB Members, Composition, Review Process | Survey | Membership, Structures and procedures, Post Approval Monitoring |
| **Arshad et al(13)** | Europe | Researchers, Review Process | Survey | IRB Decision Making, Outcome, Post Approval Monitoring |
| **Ateudjieu et al(14)** | Africa | IRB Members | Administrative Review | Training, Policy Adherence, IRB Member Views |
| **Banos et al(15)** | Europe | Health Care Workers, IRB Members | Survey | Training, Knowledge, IRB Member Views |
| **Bartlett et al(16)** | USA | Composition, Review Process | Administrative Review | Number of Protocols, Working Hours, Structures and procedures, Membership |
| **Beagan et al(17)** | Canada | IRB Members | Interviews | IRB Decision Making, IRB Member Views |
| **Berry(18)** | Europe | Research Participant | Survey | Post Approval Monitoring, Participant Views |
| **Borovecki et al(19)** | Europe | IRB Members, Composition | Survey | Membership, Knowledge, Training |
| **Borovecki et al(20)** | Europe | IRB Members, Composition, Review Process | Survey | Membership, Training, Policy Adherence, Structures and procedures, IRB Decision Making |
| **Bortolussi et al(21)** | Canada | Review Process | Administrative Review | Structures and procedures |
| **Boyce(22)** | Europe | Composition, Review Process | Administrative Review | Membership, Number of Protocols, Outcome, IRB Decision Making, Time |
| **Brahme et a(23)l** | Asia | IRB Members, Composition, Review Process | Survey | IRB Member Views, Membership, Training, Knowledge |
| **Brown et al(24)** | Europe | IRB Members | Survey | IRB Member Views |
| **Burris et al(25)** | USA | Researchers, Review Process | Interviews | Researcher Views, Working Hours, Structures and procedures |
| **Byrne et al(26)** | USA | IRB Members, Review Process | Survey | Cost, Time, Number of Protocols, Structures and procedures, Variation |
| **Campbell et al(27)** | USA | IRB Members, Review Process | Survey | Structures and procedures, IRB Member Views |
| **Catania et al(28)** | USA | Composition, Review Process | Administrative Review | IRB Member Views, Number of Protocols, Membership |
| **Carline(29)** | USA | Researchers, Health Care Workers, Review Process | Interviews | Training, Knowledge, Structures and procedures |
| **Catania et al(30)** | USA | IRB Members, Composition Review Process | Administrative Review | IRB Member Views, Number of Protocols, Membership |
| **Chakladar et al(31)** | Europe | Review Process | Administrative Review | Cost, Structures and procedures |
| **Chaudhry et al(32)** | Canada | Researchers, Health Care Workers | Survey | Researcher Views, Variation, Post Approval Monitoring, Cost, IRB Decision Making, Outcome |
| **Coker et al(33)** | Europe | Researchers | Survey | Membership, Structures and procedures, Training, Variation, Post Approval Monitoring |
| **Conforti et al(34)** | USA | Researchers | Survey | Time, Researcher Views, Structures and procedures |
| **Cook et al(35)** | Europe | Review Process | Administrative Review | Outcome, Time, Variation, IRB Decision Making |
| **Coughlin et al(36)** | Canada | IRB Members, Composition | Survey | Membership, IRB Member Views |
| **Czarkowski et al(37)** | Europe | IRB Members, Composition, Review Process | Survey | Membership, Number of Protocols, IRB Decision Making, Outcome, Working Hours, Policy Adherence |
| **Dal-Re et al(38)** | Europe | Review Process | Administrative Review | Cost, Variation, Outcome, IRB Decision Making, Structures and procedures |
| **Dal-Re et al(39)** | Europe | Review Process | Administrative Review | Structures and procedures, Membership , Time, Outcome, IRB Decision Making |
| **Davies et al(40)** | Europe | IRB Members | Workshop | IRB Member Views |
| **De Jong et al(41)** | Europe | Review Process | Administrative Review, Survey, Literature Search | Time, Outcome, Post Approval Monitoring, |
| **De Jong et al(42)** | USA | IRB Members, Review Process | Interviews | Post Approval Monitoring, Variation |
| **De Montgolfier et al(43)** | Europe | IRB Members | Survey | IRB Member Views |
| **Decullier(44)** | Europe | IRB Members, Review Process | Administrative Review | Policy Adherence, Membership, Outcome, Time, IRB Decision Making |
| **Denham et al(45)** | Europe | Review Process | Administrative Review, Longitudinal Study | Outcome |
| **Dolor et al(46)** | USA | IRB Members, Review Process | Focus Group | Training, Variation |
| **Douglass et al(47)** | Oceania | Researchers, Review Process | Interviews | Variation, Post Approval Monitoring, Researcher Views |
| **Driscoll et al(48)** | Oceania | Review Process | Administrative Review | Structures and procedures, Time, Policy Adherence, Variation, Time |
| **Druml et al(49)** | Europe | IRB Members, Composition, Review Process | Survey | Membership, Training, Structures and procedures |
| **Dunn et al(50)** | Europe | Review Process | Administrative Review | Cost, Structures and procedures |
| **Dyrbye et al(51)** | USA | Review Process | Administrative Review | Time, IRB Decision Making, Outcome, Variation, Structures and procedures |
| **Dziak et al(52)** | USA | IRB Members, Review Process | Administrative Review | IRB Decision Making, Structures and procedures, Outcome, Variation |
| **Eaton(53)** | Canada | IRB Members, Review Process | Administrative Review | IRB Decision Making |
| **Elliott et a(54)l** | Europe | IRB Members | Survey | Membership, Variation, IRB Member Views |
| **Elwyn et al(55)** | Europe | Review Process | Administrative Review | Time, Variation |
| **Eyelade et al(56)** | Africa | Review Process | Administrative Review | Time, IRB Decision Making, Outcome, Structures and procedures, Number of Protocols |
| **Ezzat et al(57)** | Canada | Review Process | Administrative Review | Time, Structures and procedures, IRB Decision Making, |
| **Falusi(58)** | Africa | Review Process | Administrative Review | Number of Protocols, Time |
| **Fauriel et al(59)** | Europe | IRB Members, Composition, Review Process | Administrative Review, Observational | Structures and procedures, Membership, Training, IRB Decision Making |
| **Feldman et al(60)** | USA | IRB Members, Review Process | Survey | Structures and procedures, Outcome, Working Hours |
| **Finch et al(61)** | USA | Review Process | Administrative Review | Time, Outcome, IRB Decision Making, Post Approval Monitoring |
| **Fitzgerald et a(62)l** | USA, Canada, Europe, Oceania | IRB Members, Review Process | Observational, Interviews | Variation, IRB Decision Making, Structures and procedures, Number of Protocols |
| **Fitzgerald et al(63)** | Oceania | IRB Members, Review Process | Observational | Variation, Structures and procedures, IRB Decision Making |
| **Flynn et al(64)** | USA | IRB Members | Literature Search, Focus Group | Researcher Views |
| **Foster et al(65)** | Europe | IRB Members, Composition, Review Process | Survey | IRB Member Views, Membership, Structures and procedures, Policy Adherence, Variation |
| **Foster et al.(66)** | Europe | IRB Members, Composition, Review Process | Survey | Membership, Outcome, IRB Decision Making, Policy Adherence, Number of Protocols, Working Hours |
| **Garfield(67)** | Europe | Review Process | Administrative Review | Structures and procedures, Outcome, IRB Decision Making, Time |
| **Geisser et al(68)** | USA | Researchers, IRB Members | Delphi Method | IRB Decision Making, IRB Member Views |
| **Gibson et al(69)** | Canada | IRB Members | Interviews | IRB Member Views, Post Approval Monitoring |
| **Gilbert et al(70)** | Europe | IRB Members, Composition, Review Process | Administrative Review, Survey | Membership, Number of Protocols, Structures and procedures |
| **Gillam et al(71)** | Oceania | Researchers, IRB Members | Interviews | IRB Decision Making, IRB Member Views |
| **Gillam et al (72)** | Oceania | Researchers, IRB Members | Survey | IRB Member Views |
| **Godfrey et al.(73)** | Europe | IRB Members, Composition, Review Process | Administrative Review | Membership, Structures and procedures, Outcome |
| **Goodyear-Smith et al(74)** | Oceania, Asia, USA, Canada, Europe | IRB Members, Review Process | Administrative Review | Variation, Structures and procedures, IRB Decision Making, |
| **Graham et al(75)** | USA | Review Process | Administrative Review | Structures and procedures, Time, Working Hours |
| **Gray et al(76)** | USA | Researchers, IRB Members, Review Process | Interviews | Outcome, IRB Decision Making, Time, Researcher Views, IRB Member Views |
| **Hannigan et al (77)** | USA | Review Process | Administrative Review | Variation, IRB Decision Making, |
| **Harries et al(78)** | Europe | Review Process | Observational | Outcome, IRB Decision Making |
| **Hayes et al(79)** | USA | IRB Members, Composition, Review Process | Survey | Membership, Number of Protocols, Training, Structures and procedures, Outcome |
| **Hearnshaw(80)** | Europe | Researchers, Review Process | Survey | IRB Decision Making, Outcome, Time, Structures and procedures, Working Hours |
| **Helfand et al(81)** | USA | Composition, Review Process | Administrative Review | Membership, Structures and procedures, Outcome, IRB Decision Making, Time |
| **Hernandez et al(82)** | Europe | IRB Members, Composition, Review Process | Survey | Membership, Structures and procedures, IRB Member Views |
| **Hirshon et al(83)** | USA | Review Process | Administrative Review | Time, Outcome, Variation, Structures and procedures, |
| **Hotopf et al(84)** | Europe | Review Process | Administrative Review | Time, IRB Member Views, IRB Decision Making |
| **Howe et al(85)** | Europe | Research Participant, IRB Members, Review Process | Administrative Review, Literature Search, Interviews, Survey, Focus Group | IRB Member Views, Structures and procedures, Membership |
| **Humphreys et al(86)** | USA | Review Process | Administrative Review | Cost |
| **Hyder et al(87)** | USA, Asia, Africa, Latin America | Researchers | Survey | Researcher Views, Membership, Time, Policy Adherence |
| **Ikingura et al(88)** | Africa | IRB Members, Composition, Review Process | Survey, Interviews | Membership, Training, Structures and procedures, Policy Adherence, Post Approval Monitoring, IRB Member Views |
| **Jones et al(89)** | USA | IRB Members, Composition, Review Process | Survey | Membership, Structures and procedures, IRB Member Views, IRB Decision Making |
| **Kallgren et al(90)** | USA | Researchers | Survey | Researcher Views |
| **Karunaratne et al(91)** | Oceania | Research Participant, IRB Members | Focus Group, Interviews, Survey | IRB Member Views, Structures and procedures, Variation, Researcher Views |
| **Kass et al(92)** | USA | Researchers, Review Process | Focus Group, Survey | Knowledge, Structures and procedures, Researcher Views, Policy Adherence |
| **Keith-Spiegel et al(93)** | USA | Researchers | Survey | Membership, IRB Member Views, Researcher Views |
| **Kent(94)** | Europe | Research Participant, Researchers, IRB Members | Survey | IRB Member Views, Researcher Views, Participant Views, Variation |
| **Kielmann et al (95)** | Europe | Review Process | Administrative Review | IRB Decision Making, Outcome, Time, Cost, Variation |
| **Kim et al(96)** | Asia | IRB Members, Composition, Review Process | Survey | Membership, Structures and procedures, Variation, Training |
| **Kimberly et al (97)** | USA | Review Process | Administrative Review | IRB Decision Making, Variation, Outcome |
| **Kirigia et al(98)** | Africa | IRB Members, Composition, Review Process | Survey | Structures and procedures, Variation, IRB Decision Making, Working Hours |
| **Klitzman(99)** | USA, Africa | IRB Members, Review Process | Survey | Structures and procedures, IRB Member Views, Variation, IRB Decision Making, Outcome |
| **Klitzman(100)** | USA | IRB Members | Interviews | IRB Member Views |
| **Klitzman(101)** | USA | IRB Members | Interviews | IRB Member Views, Structures and procedures, |
| **Klitzman(102)** | USA | IRB Members | Interviews | IRB Member Views |
| **Klitzman(103)** | USA | IRB Members | Interviews | IRB Member Views, Structures and procedures, IRB Decision Making, Training |
| **Klitzman(104)** | USA | IRB Members | Interviews | IRB Member Views |
| **Klitzman(105)** | USA | IRB Members | Interviews | IRB Member Views, Structures and procedures, Variation |
| **Klitzman(106)** | USA | IRB Members | Interviews | IRB Member Views, IRB Decision Making |
| **Koerner(107)** | USA | Researchers, Review Process | Observational | IRB Decision Making, Researcher Views |
| **Kotecha et al(108)** | Canada | Review Process | Administrative Review | Time, IRB Decision Making |
| **Larson et al(109)** | USA | IRB Members, Review Process | Administrative Review | Membership, Structures and procedures, Policy Adherence, Time |
| **Lewis et al(110)** | Europe | Review Process | Observational | Cost, Time, Working Hours, Structures and procedures, Outcome, IRB Decision Making |
| **Lidz et al.(111)** | USA | IRB Members, Review Process | Observational, Administrative Review | Policy Adherence |
| **Loh et al(112)** | USA | Researchers, Review Process | Survey | IRB Member Views, Time |
| **Lux et al(113)** | Europe | Review Process | Administrative Review | Time, Structures and procedures, Cost, Variation |
| **Lynoe et al(114)** | Europe | Researchers, Health Care Workers, IRB Members, Review Process | Survey | IRB Decision Making, Variation, Structures and procedures |
| **Malouff et al(115)** | Oceania | Researchers | Survey | Researcher Views |
| **Mammel et al(116)** | USA | IRB Members, Composition, Review Process | Survey | Membership, Structures and procedures, Variation, Policy Adherence |
| **Mansbach et al(117)** | USA | Researchers, Composition, Review Process | Survey | Structures and procedures, Membership, Time, IRB Decision Making, Outcome, IRB Member Views |
| **Maskell et al(118)** | Europe | Researchers, Review Process | Survey | Cost, Time, Structures and procedures, Working Hours, IRB Decision Making, Researcher Views |
| **McClure et al(119)** | USA | IRB Members | Interviews | IRB Member Views, Policy Adherence, Training, Structures and procedures |
| **McCusker et al(120)** | Canada | Researchers, Review Process | Survey, Administrative Review, Interviews | Outcome, Post Approval Monitoring, IRB Decision Making, |
| **McGrath et al(121)** | USA | Research Participant, Researchers, IRB Members Composition, Review Process | Interviews, Observational | Structures and procedures, Working Hours, Membership |
| **McNeill et al(122)** | Oceania | IRB Members, Composition, Review Process | Survey | Structures and procedures, Membership, IRB Decision Making, Post Approval Monitoring, Policy Adherence |
| **McNeill et al(123)** | Oceania | Researchers | Survey, Interviews | Researcher Views |
| **McNeill et al(124)** | Oceania | IRB Members, Composition | Interviews | IRB Member Views, Membership |
| **McWilliams et al(125)** | USA | Researchers, Review Process | Literature Search, Survey, Administrative Review | Structures and procedures, Time, IRB Decision Making, Outcome, Variation |
| **Middle et al(126)** | Europe | IRB Members, Review Process | Survey, Administrative Review | Outcome, IRB Decision Making, Time, Structures and procedures, Variation, Working Hours |
| **Milford et al(127)** | Africa | IRB Members, Composition, Review Process | Survey | Membership, Training, Policy Adherence, Structures and procedures, IRB Member Views |
| **Mosconi et al(128)** | Europe | Health Care Workers, Review Process | Survey | Researcher Views, Satisfaction, Time |
| **Moutel et al(129)** | Europe | IRB Members | Survey | IRB Member Views |
| **National Council on Ethics in Human Research(130)** | Canada | Composition, Review Process | Observational | IRB Member Views |
| **Nelson et al(131)** | USA | Research Participant, Review Process | Administrative Review | Structures and procedures, Variation, IRB Decision Making, Outcome |
| **Nilstun et al(132)** | Europe, Asia | Researchers, Review Process | Survey | Policy Adherence, Structures and procedures, Variation, IRB Decision Making |
| **Norton et al(133)** | Canada | IRB Members, Review Process | Survey | Post Approval Monitoring, Structures and procedures, Variation |
| **Nyika et al(134)** | Africa | IRB Members, Composition, Review Process | Survey | Membership, Cost , Structures and procedures |
| **O'Herrin et al(135)** | USA | Review Process | Administrative Review | IRB Decision Making, Time, Outcome, Structures and procedures, Post Approval Monitoring |
| **Olsen et al(136)** | USA | Review Process | Administrative Review | IRB Decision Making |
| **Panichkul et al(137)** | Asia | IRB Members, Composition, Review Process | Survey | Membership, Structures and procedures, Post Approval Monitoring, IRB Member Views |
| **Patel et al(138)** | USA | Review Process | Administrative Review | Outcome, Structures and procedures, IRB Decision Making, Time |
| **Paul(139)** | Oceania | Researchers, Review Process | Survey | Researcher Views |
| **Pehboeck et al(140)** | Europe | Researchers, Review Process | Survey | Membership, Number of Protocols, Post Approval Monitoring, Researcher Views |
| **Pich et al(141)** | Europe | Review Process | Administrative Review | Outcome |
| **Porcu et al (142)** | Europe | Review Process | Administrative Review | Structures and procedures, Time, Variation |
| **Ravina et al(143)** | USA | Review Process | Administrative Review | Variation, Working Hours, Cost |
| **Redshaw et al(144)** | USA | Review Process | Administrative Review | Structures and procedures, Variation, Time |
| **Reeser et al(145)** | USA | Researchers, IRB Members | Survey | IRB Member Views, Researcher Views, Membership, |
| **Rikkert et al (146)** | Europe | IRB Members, Review Process | Survey | IRB Decision Making, Outcome, Structures and procedures, Variation |
| **Rogers et al(147)** | USA | Researchers, Review Process | Administrative Review, Survey | Researcher Views, Number of Protocols, Structures and procedures, IRB Decision Making |
| **Rothstein et al(148)** | USA | IRB Members, Composition | Survey | IRB Member Views |
| **Russ et al(149)** | Europe | IRB Members, Review Process | Survey | IRB Decision Making, Outcome |
| **Saginur et al(150)** | Canada | Review Process | Administrative Review | Number of Protocols, Structures and procedures, Time |
| **Saito(151)** | Asia | IRB Members, Composition, Review Process | Survey | Membership, Structures and procedures, IRB Member Views, Variation, Post Approval Monitoring |
| **Sarpel et al (152)** | USA | Health Care Workers | Administrative Review, Focus Group, Survey | Variation, Participant Views, Outcome |
| **Schellings et al(153)** | Europe | IRB Members, Review Process | Survey | Structures and procedures, Variation, Outcome |
| **Schick et al(154)** | USA | IRB Members, Composition, Review Process | Survey | Membership, Variation, IRB Member Views, Satisfaction |
| **Sengupta et al (155)** | USA | IRB Members | Interviews | Training, IRB Member Views |
| **Shah et al(156)** | USA | IRB Members | Administrative Review | Variation |
| **Sherwood et al(157)** | USA | Review Process | Administrative Review | Time, Structures and procedures, IRB Decision Making |
| **Silberman et al(158)** | USA | Review Process | Literature Search | Cost, IRB Decision Making, Working Hours, Variation, Outcome, Policy Adherence, Structures and procedures |
| **Silverman et al(159)** | USA | IRB Members, Review Process | Survey | Structures and procedures, Variation |
| **Simek et al(160)** | Europe | Researchers, IRB Members, Composition, Review Process, Membership | Observational, Focus Group, Survey | IRB Member Views, Structures and procedures, Membership, Knowledge |
| **Skrutkowski et al(161)** | Canada | Research Participant | Interviews | Participant Views, Working Hours |
| **Sleem et al(162)** | Africa, Asia | IRB Members | Survey | IRB Member Views |
| **Sleem et al(163)** | Africa | IRB Members, Composition, Review Process | Survey | Membership, Training, Working Hours, Number of Protocols, Structures and procedures, IRB Member Views, Cost |
| **Smith et al(164)** | Europe | Review Process | Administrative Review | Time, Structures and procedures |
| **Speckman et al (165)** | USA | IRB Members, Composition, Review Process | Administrative Review, Survey, Observational | Membership, Cost, Structures and procedures, Post Approval Monitoring |
| **Stair et al(166)** | USA | Researchers, IRB Members, Composition, Review Process | Survey | Working Hours, Membership, Structures and procedures, Time, IRB Decision Making, Outcome |
| **Stark et al(167)** | USA | Review Process | Administrative Review | IRB Decision Making, Variation |
| **Sugarman et al(168)** | USA | Review Process | Survey, Interviews | Membership, Time, Structures and procedures |
| **Sumathipala et al(169)** | Asia | IRB Members, Review Process | Interviews, Survey | Structures and procedures, Variation, Policy Adherence, IRB Member Views |
| **Taylor et al(170)** | USA | Review Process | Administrative Review | Time, Structures and procedures |
| **Taylor et al(171)** | USA | Review Process | Administrative Review | Structures and procedures, Variation, IRB Decision Making, Outcome |
| **Thompson et al(172)** | Europe | IRB Members | Survey | IRB Member Views, Membership, Variation, Structures and procedures |
| **Thompson et al(173)** | USA | Review Process | Administrative Review | Structures and procedures, Time |
| **Tsan et al(174)** | USA | Researchers, IRB Members | Focus Group | IRB Member Views |
| **Tsan et al(175)** | USA | Review Process | Administrative Review | Variation, Policy Adherence, Structures and procedures, Training, Post Approval Monitoring |
| **Tsan et al (176)** | USA | Review Process | Administrative Review | Structures and procedures, Variation, Outcome, IRB Decision Making, Post Approval Monitoring |
| **Tully et al(177)** | Europe | Review Process | Administrative Review | Structures and procedures, Time, Cost, IRB Decision Making |
| **Valdez-Martinez et al (178)** | Latin America | Review Process | Administrative Review | Membership, Structures and procedures, Post Approval Monitoring |
| **Valdez-Martinez et al (179)** | Latin America | IRB Members, Review Process | Focus Group | IRB Member Views, Knowledge |
| **Van Essen et al (180)** | Oceania | IRB Members, Review Process | Survey | Structures and procedures, Membership |
| **Van Luijn et al (181)** | Europe | IRB Members, Review Process | Survey | IRB Member Views |
| **Vick et al (182)** | USA | Review Process | Administrative Review | Time, Cost, Variation Working Hours, IRB Decision Making |
| **Vogeli et al (183)** | USA | IRB Members, Review Process | Survey | Structures and procedures, Membership, IRB Member Views |
| **Vulcano(184)** | USA | IRB Members | Survey | Membership, IRB Member Views, Policy Adherence, IRB Decision Making |
| **Vulcano(185)** | USA | Composition | Administrative Review | Membership, Number of RECs in Region, Number of Protocols |
| **Wagner et al (186)** | USA | Review Process | Administrative Review | Cost |
| **Wagner et al(187)** | USA | IRB Members | Survey | Cost |
| **Wagner et al(188)** | USA | IRB Members ,Review Process | Survey | Working Hours, Time, Cost, Variation |
| **Watson et al (189)** | Oceania | Review Process | Administrative Review | Structures and procedures, Cost, Variation, Time, IRB Decision Making, Post Approval Monitoring |
| **Whitney et al(190)** | USA | Researchers, Review Process | Survey | Researcher Views |
| **Wichman et al (191)** | USA | Review Process | Survey | Policy Adherence, IRB Decision Making, Outcome, Knowledge, Structures and procedures |
| **Williams-Jones et al (192)** | Canada | IRB Members | Interviews | Structures and procedures, IRB Members Views |
| **Willison et al (193)** | Canada | IRB Members | Interviews | IRB Member Views |
| **Wisner et al(194)** | USA | Researchers, Review Process | Survey | Researcher Views |
| **Wu et al (195)** | Asia | Health Care Workers, Composition | Literature Search, Focus Group, Delphi Method, Survey | Membership, IRB Member Views |
| **Yawn et al (196)** | USA | Review Process | Administrative Review | Structures and procedures, Variation, Time, Training |
| **Zeeneldin(197)** | Africa | Review Process | Administrative Review | Variation, Outcome |
| **Zhou et al (198)** | Asia | IRB Members, Composition, Review Process | Survey, Interviews, Administrative Review | Structures and procedures, Membership |

Reference List

(1) Abbott L, Grady C. A systematic review of the empirical literature evaluating IRBs: what we know and what we still need to learn. [Review]. Journal of Empirical Research on Human Research Ethics 2011 Mar;6(1):3-19.

(2) Abou-Zeid A, Afzal M, Silverman HJ. Capacity mapping of national ethics committees in the Eastern Mediterranean Region. BMC Medical Ethics 2009;10:8.

(3) Ahmed AH, Nicholson KG. Delays and diversity in the practice of local research ethics committees. Journal of Medical Ethics 1996;22(5):263-6.

(4) Ah-See KW, MacKenzie J, Thakker NS, Maran AG. Local research ethics committee approval for a national study in Scotland. Journal of the Royal College of Surgeons of Edinburgh 1998 Oct;43(5):303-5.

(5) Akabayashi A, Slingsby BT, Nagao N, Kai I, Sato H. An eight-year follow-up national study of medical school and general hospital ethics committees in Japan. BMC Medical Ethics 2007;8:8.

(6) Allen HJ. Genetic protocols review by Institutional Review Boards at National Cancer Institute-designated cancer centers. Genetic Testing 1998;2(4):329-36.

(7) Allen PA, Waters WE. Development of An Ethical Committee and Its Effect on Research Design. Lancet 1982;1(8283):1233-6.

(8) Allen P, Waters WE. Attitudes to research ethical committees. Journal of Medical Ethics 1983 Jun;9(2):61-5.

(9) Al-Shahi R, Warlow CP. Ethical review of a multicentre study in Scotland: a weighty problem. Journal of the Royal College of Physicians of London 1999;33(6):549-52.

(10) Angell E, Sutton A, Windridge K, Dixon-Woods M. Consistency in decision making by research ethics committees: a controlled comparison. Journal of Medical Ethics 2006;32(11):662-4.

(11) Angell EL, Bryman A, Ashcroft RE, Dixon-Woods M. An analysis of decision letters by research ethics committees: the ethics/scientific quality boundary examined. Quality & Safety in Health Care 2008 Apr;17(2):131-6.

(12) Arda B. The experience of the research ethics committees in Turkey. Medicine & Law 2000;19(3):493-500.

(13) Arshad A, Arkwright PD. Status of healthcare studies submitted to UK research ethics committees for approval in 2004-5. Journal of Medical Ethics 2008 May;34(5):393-5.

(14) Ateudjieu J, Williams J, Hirtle M, Baume C, Ikingura J, Niare A, et al. Training needs assessment in research ethics evaluation among research ethics committee members in three African countries: Cameroon, Mali and Tanzania. Developing World Bioethics 2010 Aug;10(2):88-98.

(15) Banos JE, Lucena MI, Seres E, Bosch F. Reflections on running training workshops for research ethics committee members in Spain between 2001 and 2008. Croatian Medical Journal 2010 Dec;51(6):552-9.

(16) Bartlett EE. International Analysis of Institutional Review Boards Registered with the Us Office for Human Research Protections. Journal of Empirical Research on Human Research Ethics 2008;3(4):49-56.

(17) Beagan B, McDonald M. Evidence-based practice of research ethics review? Health law review 2005;13(2-3):62-8.

(18) Berry J. Local Research Ethics Committees can audit ethical standards in research. Journal of Medical Ethics 1997 Dec;23(6):379-81.

(19) Borovecki A, ten HH, Oreskovic S. Education of ethics committee members: experiences from Croatia. Journal of Medical Ethics 2006 Mar;32(3):138-42.

(20) Borovecki A, Oreskovic S, ten HH. Ethics and the structures of health care in the European countries in transition: hospital ethics committees in Croatia. BMJ 2005 Jul 23;331(7510):227-9.

(21) Bortolussi R, Nicholson D. Auditing of clinical research ethics in a children's and women's academic hospital. Clinical and Investigative Medicine-Medecine Clinique et Experimentale 2002;25(3):83-8.

(22) Boyce M. Observational study of 353 applications to London multicentre research ethics committee 1997-2000. British Medical Journal 2002;325(7372):1081.

(23) Brahme R, Mehendale S. Profile and role of the members of ethics committees in hospitals and research organisations in Pune, India. Indian Journal of Medical Ethics 2009 Apr;6(2):78-84.

(24) Brown J, Ryland I, Howard J, Shaw N. Views of National Health Service (NHS) Ethics Committee members on how education research should be reviewed. Medical Teacher 2007 Mar;29(2-3):225-30.

(25) Burris S, Moss K. U. S. health researchers review their ethics review boards: A qualitative study. Journal of Empirical Research on Human Research Ethics 2006;1(2):39-58.

(26) Byrne MM, Speckman J, Getz K, Sugarman J. Variability in the costs of institutional review board oversight. Academic Medicine 2006 Aug;81(8):708-12.

(27) Campbell EG, Weissman JS, Vogeli C, Clarridge BR, Abraham M, Marder JE, et al. Financial relationships between institutional review board members and industry. New England Journal of Medicine 2006 Nov 30;355(22):2321-9.

(28) Catania JA, Lo B, Wolf LE, Dolcini MM, Pollack LM, Barker JC, et al. Survey of u.s. Human research protection organizations: workload and membership. Journal of Empirical Research on Human Research Ethics 2008 Dec;3(4):57-69.

(29) Carline JD, O'Sullivan PS, Gruppen LD, Richardson-Nassif K. Crafting Successful Relationships with the IRB. Acad Med 2007 Oct;82(10 Suppl):S57-S60.

(30) Catania JA, Lo B, Wolf LE, Dolcini MM, Pollack LM, Barker JC, et al. Survey of U.S. Boards that Review Mental Health-related Research. Journal of Empirical Research on Human Research Ethics 2008 Dec;3(4):71-9.

(31) Chakladar A, Eckstein S, White SM. Paper use in research ethics applications and study conduct. Clinical Medicine 2011 Feb;11(1):44-7.

(32) Chaudhry SH, Brehaut JC, Grimshaw JM, Weijer C, Boruch R, Donner A, et al. Challenges in the research ethics review of cluster randomized trials: international survey of investigators. Clinical Trials 2013 Apr;10(2):257-68.

(33) Coker R, McKee M. Ethical approval for health research in central and eastern Europe: an international survey. Clinical Medicine 2001 May;1(3):197-9.

(34) Conforti LN, Hess BJ, Ross KM, Lynn LA, Holmboe ES. Variability in obtaining institutional review board approval for quality improvement activities in residency programs. Journal of Graduate Medical Education 2012 Mar;4(1):106-8.

(35) Cook M, Cook G, Hodgson P, Reed J, Clarke C, Inglis P. The impact of research governance in the United Kingdom on research involving a national survey. Journal of Health Organization & Management 2007;21(1):59-67.

(36) Coughlin MD, Watts J. A descriptive study of healthcare ethics consultants in Canada: results of a national survey. HEC Forum 1993 May;5(3):144-64.

(37) Czarkowski M, Rozanowski K. Polish Research Ethics Committees in the European Union system of assessing medical experiments. Science & Engineering Ethics 2009 Jun;15(2):201-12.

(38) Dal-Re R, Morejon E, Ortega R. Nature and extent of changes in the patient's information sheets of international multicentre clinical trials as requested by Spanish Research Ethics Committees. Medicina Clinica 2004 Dec 4;123(20):770-4.

(39) Dal-Re R, Espada J, Ortega R. Performance of research ethics committees in Spain. A prospective study of 100 applications for clinical trial protocols on medicines. Journal of Medical Ethics 1999;25(3):268-73.

(40) Davies H, Wells F, Czarkowski M. Standards for research ethics committees: purpose, problems and the possibilities of other approaches. Journal of Medical Ethics 2009;35(6):382-3.

(41) de Jong JP, Ter RG, Willems DL. Two prognostic indicators of the publication rate of clinical studies were available during ethical review. Journal of Clinical Epidemiology 2010 Dec;63(12):1342-50.

(42) de Jong JP, van Zwieten MC, Willems DL. Research monitoring by US medical institutions to protect human subjects: compliance or quality improvement? Journal of Medical Ethics 2013 Apr;39(4):236-41.

(43) de MS, Moutel G, Duchange N, Callies I, Sharara L, Beaumont C, et al. Evaluation of biobank constitution and use: multicentre analysis in France and propositions for formalising the activities of research ethics committees. European Journal of Medical Genetics 2006 Mar;49(2):159-67.

(44) Decullier E, Lh+¬ritier V+, Chapuis F. The activity of French Research Ethics Committees and characteristics of biomedical research protocols involving humans: a retrospective cohort study. BMC Med Ethics 2005;6(1):1-10.

(45) Denham MJ, Foster A, Tyrrell DAJ. Work of A District Ethical Committee. British Medical Journal 1979;2(6197):1042-5.

(46) Dolor RJ, Smith PC, Neale AV, Agency for Health Care Research and Quality Practice-Based Research Network. Institutional review board training for community practices: advice from the Agency for Health Care Research and Quality Practice-Based Research Network listserv. [Review] [16 refs]. Journal of the American Board of Family Medicine: JABFM 2008 Jul;21(4):345-52.

(47) Douglass AJ, Jarvis A, Bloore S. Monitoring of health research by ethics committees. New Zealand Medical Journal 1998;111(1061):79-81.

(48) Driscoll A, Currey J, Worrall-Carter L, Stewart S. Ethical dilemmas of a large national multi-centre study in Australia: time for some consistency. Journal of Clinical Nursing 2008 Aug;17(16):2212-20.

(49) Druml C, Wolzt M, Pleiner J, Singer E. Research ethics committees in Europe: trials and tribulations. Intensive Care Medicine 2009;35(9):1636-40.

(50) Dunn NR, Arscott A, Mann RD. Costs of seeking ethics approval before and after the introduction of multicentre research ethics committees. Journal of the Royal Society of Medicine 2000 Oct;93(10):511-2.

(51) Dyrbye LN, Thomas MR, Mechaber AJ, Eacker A, Harper W, Massie FS, Jr., et al. Medical education research and IRB review: an analysis and comparison of the IRB review process at six institutions. Academic Medicine 2007 Jul;82(7):654-60.

(52) Dziak K, Anderson R, Sevick MA, Weisman CS, Levine DW, Scholle SH. Variations among Institutional Review Board reviews in a multisite health services research study. Health Services Research 2005 Feb;40(1):279-90.

(53) Eaton WO. Reliability in Ethics Reviews - Some Initial Empirical-Findings. Canadian Psychology-Psychologie Canadienne 1983;24(1):14-8.

(54) Elliott L, Hunter D. The experiences of ethics committee members: contradictions between individuals and committees. Journal of Medical Ethics 2008 Jun;34(6):489-94.

(55) Elwyn G, Seagrove A, Thorne K, Cheung WY. Ethics and research governance in a multicentre study: add 150 days to your study protocol. BMJ 2005 Apr 9;330(7495):847.

(56) Eyelade OR, Ajuwon AJ, Adebamowo CA. An appraisal of the process of protocol review by an ethics review conmmittee in a tertiary institution in Ibadan. African Journal of Medicine & Medical Sciences 2011 Jun;40(2):163-9.

(57) Ezzat H, Ross S, von DP, Morris T, Liston R, Magee LA, et al. Ethics review as a component of institutional approval for a multicentre continuous quality improvement project: the investigator's perspective. BMC Health Services Research 2010;10:223.

(58) Falusi AG, Olopade OI, Olopade CO. Establishment of a standing ethics/institutional review board in a nigerian university: a blueprint for developing countries. J Empir Res Hum Res Ethics 2007 Mar;2(1):21-30.

(59) Fauriel I, Moutel G, Duchange N, Callies I, Francois I, Huriet C, et al. Improving protection for research subjects in France: analysis of regional ethics committees. Regulatory Toxicology and Pharmacology 2004;40(3):312-8.

(60) Feldman JA, Rebholz CM. Anonymous self-evaluation of performance by ethics board members: a pilot study. Journal of Empirical Research on Human Research Ethics 2009 Mar;4(1):63-9.

(61) Finch SA, Barkin SL, Wasserman RC, Dhepyasuwan N, Slora EJ, Sege RD. Effects of Local Institutional Review Board Review on Participation in National Practice-Based Research Network Studies. Archives of Pediatrics & Adolescent Medicine 2009;163(12):1130-4.

(62) Fitzgerald MH, Phillips PA. Centralized and non-centralized ethics review: a five nation study. Accountability in research 2006;13(1):47-74.

(63) Fitzgerald MH, Phillips PA, Yule E. The research ethics review process and ethics review narratives. Ethics & Behavior 2006;16(4):377-95.

(64) Flynn KE, Hahn CL, Kramer JM, Check DK, Dombeck CB, Bang S, et al. Using central IRBs for multicenter clinical trials in the United States. PLoS ONE [Electronic Resource] 2013;8(1):e54999.

(65) Foster C, Holley S. Ethical review of multi-centre research: a survey of multi-centre researchers in the South Thames region. Journal of the Royal College of Physicians of London 1998 May;32(3):242-5.

(66) Foster CG, Marshall T, Moodie P. The Annual-Reports of Local Research Ethics Committees. Journal of Medical Ethics 1995;21(4):214-9.

(67) Garfield P. Cross District Comparison of Applications to Research Ethics Committees. British Medical Journal 1995;311(7006):660-1.

(68) Geisser ME, Alschuler KN, Hutchinson R. A delphi study to establish important aspects of ethics review. Journal of Empirical Research on Human Research Ethics 2011 Mar;6(1):21-4.

(69) Gibson E, Brazil K, Coughlin MD, Emerson C, Fournier F, Schwartz L, et al. Who's minding the shop? The role of Canadian research ethics boards in the creation and uses of registries and biobanks. BMC Medical Ethics 2008;9:17.

(70) Gilbert C, Fulford KWM, Parker C. Diversity in the Practice of District Ethics Committees. British Medical Journal 1989;299(6713):1437-9.

(71) Gillam L, Guillemin M, Bolitho A, Rosenthal D. Human research ethics in practice: deliberative strategies, processes and perceptions. Monash Bioethics Review 2009 Mar;28(1):7-17.

(72) Gillam L, Guillemin M, Rosenthal D. 'Obstructive and power hungry'?: the Australian human research ethics process. Monash Bioethics Review 2006;25(2):30-7.

(73) Godfrey E, Wray E, Nicholson R. Another look at LREC annual reports. Bull Med Ethics 2001 Sep;(171):13-21.

(74) Goodyear-Smith F, Lobb B, Davies G, Nachson I, Seelau SM. International variation in ethics committee requirements: comparisons across five Westernised nations. BMC Medical Ethics 2002 Apr 19;3:E2.

(75) Graham DG, Pace W, Kappus J, Holcomb S, Galliher JM, Duclos CW, et al. Institutional Review Board Approval of Practice-based Research Network Patient Safety Studies. 2005 Feb.

(76) Gray B, Cooke RA. Ethics and Regulation - Impact of Institutional Review Boards on Research. Hastings Center Report 1980;10(1):36-41.

(77) Hannigan B, Allen D. A tale of two studies: research governance issues arising from two ethnographic investigations into the organisation of health and social care. International Journal of Nursing Studies 2003 Sep;40(7):685-95.

(78) Harries UJ, Fentem PH, Tuxworth W, Hoinville GW. Local Research Ethics Committees - Widely Differing Responses to A National Survey Protocol. Journal of the Royal College of Physicians of London 1994;28(2):150-4.

(79) Hayes GJ, Hayes SC, Dykstra T. A survey of university institutional review boards: characteristics, policies, and procedures. Irb 1995;17(3):1-6.

(80) Hearnshaw H. Comparison of requirements of research ethics committees in 11 European countries for a non-invasive interventional study. BMJ 2004 Jan 17;328(7432):140-1.

(81) Helfand BT, Mongiu AK, Roehrborn CG, Donnell RF, Bruskewitz R, Kaplan SA, et al. Variation in Institutional Review Board Responses to a Standard Protocol for a Multicenter Randomized, Controlled Surgical Trial. Journal of Urology 2009;181(6):2674-9.

(82) Hernandez R, Cooney M, Duale C, Galvez M, Gaynor S, Kardos G, et al. Harmonisation of ethics committees' practice in 10 European countries. Journal of Medical Ethics 2009;35(11):696-700.

(83) Hirshon JM, Krugman SD, Witting MD, Furuno JP, Limcangco MR, Perisse AR, et al. Variability in institutional review board assessment of minimal-risk research. Academic Emergency Medicine 2002 Dec;9(12):1417-20.

(84) Hotopf M, Wessely S, Noah N. Are Ethical Committees Reliable. Journal of the Royal Society of Medicine 1995;88(1):31-3.

(85) Howe A, Delaney S, Romero J, Tinsley A, Vicary P. Public involvement in health research: a case study of one NHS project over 5 years. [References]. Primary Health Care Research and Development 2010 Jan;(1):17-28.

(86) Humphreys K, Trafton J, Wagner TH. The cost of institutional review board procedures in Multicenter Observational Research. Annals of Internal Medicine 2003;139(1):77.

(87) Hyder AA, Wali SA, Khan AN, Teoh NB, Kass NE, Dawson L. Ethical review of health research: a perspective from developing country researchers. Journal of Medical Ethics 2004 Feb;30(1):68-72.

(88) Ikingura JK, Kruger M, Zeleke W. Health research ethics review and needs of institutional ethics committees in Tanzania. Tanzania Health Research Bulletin 2007 Sep;9(3):154-8.

(89) Jones JS, White LJ, Pool LC, Dougherty JM. Structure and practice of institutional review boards in the United States. Academic Emergency Medicine 1996 Aug;3(8):804-9.

(90) Kallgren CA, Tauber RT. Undergraduate research and the institutional review board: A mismatch or happy marriage? Teaching of Psychology 1996 Feb;(1):20-5.

(91) Karunaratne AS, Myles PS, Ago MJ, Komesaroff PA. Communication deficiencies in research and monitoring by ethics committees. Internal Medicine Journal 2006 Feb;36(2):86-91.

(92) Kass N, Dawson L, Loyo-Berrios NI. Ethical Oversight of Research in Developing Countries. [References]. IRB: Ethics & Human Research 2003 Mar;(2):1-10.

(93) Keith-Spiegel P, Tabachnick B. What scientists want from their research ethics committee. Journal of Empirical Research on Human Research Ethics 2006 Mar;1(1):67-82.

(94) Kent G. The views of members of Local Research Ethics Committees, researchers and members of the public towards the roles and functions of LRECs. Journal of Medical Ethics 1997 Jun;23(3):186-90.

(95) Kielmann T, Tierney A, Porteous R, Huby G, Sheikh A, Pinnock H. The Department of Health's research governance framework remains an impediment to multi-centre studies: findings from a national descriptive study. Journal of the Royal Society of Medicine 2007;100(5):234-8.

(96) Kim OJ, Park BJ, Sohn DR, Lee SM, Shin SG. Current status of the institutional review boards in Korea: constitution, operation, and policy for protection of human research participants. Journal of Korean Medical Science 2003 Feb;18(1):3-10.

(97) Kimberly MB, Hoehn KS, Feudtner C, Nelson RM, Schreiner M. Variation in standards of research compensation and child assent practices: A comparison of 69 institutional review board-approved informed permission and assent forms for 3 multicenter pediatric clinical trials. Pediatrics 2006;117(5):1706-11.

(98) Kirigia JM, Wambebe C, Baba-Moussa A. Status of national research bioethics committees in the WHO African region. BMC Medical Ethics 2005 Oct 20;6:E10.

(99) Klitzman R. Views of the process and content of ethical reviews of HIV vaccine trials among members of US institutional review boards and South African research ethics committees. Developing World Bioethics 2008 Dec;8(3):207-18.

(100) Klitzman R. The ethics police?: IRBs' views concerning their power. PLoS ONE [Electronic Resource] 2011;6(12):e28773.

(101) Klitzman R. How local IRBs view central IRBs in the US. BMC Medical Ethics 2011;12:13.

(102) Klitzman R, Klitzman R. The Myth of Community Differences as the Cause of Variations Among IRBs. AJOB Primary Research 2011;2(2):24-33.

(103) Klitzman R. From anonymity to "open doors": IRB responses to tensions with researchers. BMC Research Notes 2012;5:347.

(104) Klitzman RL. US IRBs confronting research in the developing world. Developing World Bioethics 2012 Aug;12(2):63-73.

(105) Klitzman R. "Members of the same club": challenges and decisions faced by US IRBs in identifying and managing conflicts of interest. PLoS ONE [Electronic Resource] 2011;6(7):e22796.

(106) Klitzman R. How good does the science have to be in proposals submitted to Institutional Review Boards? An interview study of Institutional Review Board personnel. Clinical Trials 2013 Oct;10(5):761-6.

(107) Koerner AF. Communication scholars' communication and relationship with their IRBs. Journal of Applied Communication Research 2005;33(3):231-41.

(108) Kotecha JA, Manca D, Lambert-Lanning A, Keshavjee K, Drummond N, Godwin M, et al. Ethics and privacy issues of a practice-based surveillance system: need for a national-level institutional research ethics board and consent standards. Canadian Family Physician 2011 Oct;57(10):1165-73.

(109) Larson E, Bratts T, Zwanziger J, Stone P. A survey of IRB process in 68 U.S. hospitals. Journal of Nursing Scholarship 2004;36(3):260-4.

(110) Lewis JC, Tomkins S, Sampson JR. Ethical approval for research involving geographically dispersed subjects: unsuitability of the UK MREC/LREC system and relevance to uncommon genetic disorders. Journal of Medical Ethics 2001;27(5):347-51.

(111) Lidz CW, Appelbaum PS, Arnold R, Candilis P, Gardner W, Myers S, et al. How closely do institutional review boards follow the common rule? Acad Med 2012 Jul;87(7):969-74.

(112) Loh ED, Meyer RE. Medical schools' attitudes and perceptions regarding the use of central institutional review boards. Academic Medicine 2004;79(7):644-51.

(113) Lux AL, Edwards SW, Osborne JP. Responses of local research ethics committees to a study with approval from a multicentre research ethics committee. British Medical Journal 2000;320(7243):1182-3.

(114) Lynoe N, Sandlund M, Jacobsson L. Research ethics committees: a comparative study of assessment of ethical dilemmas. Scandinavian Journal of Public Health 1999 Jun;27(2):152-9.

(115) Malouff JM, Schutte NS. Academic psychologists' perspectives on the human research ethics review process. Australian Psychologist 2005;40(1):57-62.

(116) Mammel KA, Kaplan DW. Research consent by adolescent minors and institutional review boards. Journal of Adolescent Health 1995 Nov;17(5):323-30.

(117) Mansbach J, Acholonu U, Clark S, Camargo CA. Variation in institutional review board - Responses to a standard, observational, pediatric research protocol. Academic Emergency Medicine 2007;14(4):377-80.

(118) Maskell NA, Jones EL, Davies RJO. Variations in experience in obtaining local ethical approval for participation in a multi-centre study. Qjm-An International Journal of Medicine 2003;96(4):305-7.

(119) McClure KB, Delorio NM, Schmidt TA, Chiodo G, Gorman P. A qualitative study of institutional review board members' experience reviewing research proposals using emergency exception from informed consent. Journal of Medical Ethics 2007;33(5):289-93.

(120) McCusker J, Kruszewski Z, Lacey B, Schiff B. Monitoring clinical research: report of one hospital's experience. CMAJ Canadian Medical Association Journal 2001 May 1;164(9):1321-5.

(121) McGrath MM, Fullilove RE, Kaufman MR, Wallace R, Fullilove MT. The limits of collaboration: a qualitative study of community ethical review of environmental health research. American Journal of Public Health 2009 Aug;99(8):1510-4.

(122) McNeill PM, Berglund CA, Webster IW. Reviewing the Reviewers - A Survey of Institutional Ethics Committees in Australia. Medical Journal of Australia 1990;152(6):289-&.

(123) McNeill PM, Berglund CA, Webster IW. Do Australian researchers accept committee review and conduct ethical research? Soc Sci Med 1992 Aug;35(3):317-22.

(124) McNeill PM, Berglund CA, Webster IW. How much influence do various members have within research ethics committees? Cambridge Quarterly of Healthcare Ethics 1994;3(4):522-32.

(125) McWilliams R, Hoover-Fong J, Hamosh A, Beck S, Beaty T, Cutting G. Problematic variation in local institutional review of a multicenter genetic epidemiology study. Jama-Journal of the American Medical Association 2003;290(3):360-6.

(126) Middle C, Johnson A, Petty T, Sims L, Macfarlane A. Ethics Approval for A National Postal Survey - Recent Experience. British Medical Journal 1995;311(7006):659-60.

(127) Milford C, Wassenaar D, Slack C. Resources and needs of research ethics committees in Africa: Preparations for HIV vaccine trials. [References]. IRB: Ethics & Human Research 2006 Mar;(2):1-9.

(128) Mosconi P, Colombo C, Labianca R, Apolone G. Oncologists' opinions about research ethics committees in Italy: an update, 2004. European Journal of Cancer Prevention 2006 Feb;15(1):91-4.

(129) Moutel G, de MS, Duchange N, Sharara L, Beaumont C, Herve C. Study of the involvement of research ethics committees in the constitution and use of biobanks in France. Pharmacogenetics 2004 Mar;14(3):195-8.

(130) National Council on Ethics in Human Research. Report on Site Visits. October 1998 to December 2001 (Canadian Report). 2002.

(131) Nelson K, Garcia RE, Brown J, Mangione CM, Louis TA, Keeler E, et al. Do patient consent procedures affect participation rates in health services research? Medical Care 2002 Apr;40(4):283-8.

(132) Nilstun T, Cartwright C, Lofmark R, Deliens L, Fischer S, Miccinesi G, et al. Access to death certificates: what should research ethics committees require for approval? Annals of Epidemiology 2006 Apr;16(4):281-4.

(133) Norton K, Wilson DM. Continuing ethics review practices by Canadian research ethics boards. Irb: a Review of Human Subjects Research 2008 May;30(3):10-4.

(134) Nyika A, Kilama W, Chilengi R, Tangwa G, Tindana P, Ndebele P, et al. Composition, training needs and independence of ethics review committees across Africa: are the gate-keepers rising to the emerging challenges? J Med Ethics 2009 Mar;35(3):189-93.

(135) O'Herrin JK, Fost N, Kudsk KA. Health Insurance Portability Accountability Act (HIPAA) regulations: effect on medical record research. Annals of Surgery 776 Aug;239(6):772-6.

(136) Olsen DP, Mahrenholz D. IRB-identified ethical issues in nursing research. J Prof Nurs 2000 May;16(3):140-8.

(137) Panichkul S, Mahaisavariya P, Morakote N, Condo S, Caengow S, Ketunpanya A. Current status of the research ethics committees in Thailand. Journal of the Medical Association of Thailand 2011 Aug;94(8):1013-8.

(138) Patel DI, Stevens KR, Puga F. Variations in institutional review board approval in the implementation of an improvement research study. Nursing Research and Practice 2013;2013:548591.

(139) Paul C. Health researchers' views of ethics committee functioning in New Zealand. New Zealand Medical Journal 2000 Jun 9;113(1111):210-4.

(140) Pehboeck D, Hohlrieder M, Wenzel V, Benzer A. Submission of clinical studies to ethics committees or clinical trials registers: the authors' point of view. Intensive Care Medicine 2009 Apr;35(4):713-6.

(141) Pich J, Carne X, Arnaiz JA, Gomez B, Trilla A, Rodes J. Role of a research ethics committee in follow-up and publication of results. Lancet 2003;361(9362):1015-6.

(142) Porcu L, Poli D, Torri V, Rulli E, Di Tullio M, Cinquini M, et al. Impact of recent legislative bills regarding clinical research on Italian ethics committee activity. Journal of Medical Ethics 2008;34(10):747-50.

(143) Ravina B, Deuel L, Siderowf A, Dorsey ER. Local Institutional Review Board (IRB) Review of a Multicenter Trial: Local Costs without Local Context. Annals of Neurology 2010;67(2):258-60.

(144) Redshaw ME, Harris A, Baum JD. Research ethics committee audit: Differences between committees. Journal of Medical Ethics 1996;22(2):78-82.

(145) Reeser JC, Austin DM, Jaros LM, Mukesh BN, McCarty CA. Investigating Perceived Institutional Review Board Quality and Function Using the IRB Researcher Assessment Tool. Journal of Empirical Research on Human Research Ethics 2008 Mar;3(1):25-34.

(146) Rikkert MG, Lauque S, Frolich L, Vellas B, Dekkers W. The practice of obtaining approval from medical research ethics committees: a comparison within 12 European countries for a descriptive study on acetylcholinesterase inhibitors in Alzheimer's dementia. European Journal of Neurology 2005 Mar;12(3):212-7.

(147) Rogers AS, Schwartz DF, Weissman G, English A. A case study in adolescent participation in clinical research: eleven clinical sites, one common protocol, and eleven IRBs. Irb 1999;21(1):6-10.

(148) Rothstein WG, Phuong LH. Ethical attitudes of nurse, physician, and unaffiliated members of institutional review boards. Journal of Nursing Scholarship 2007;39(1):75-81.

(149) Russ H, Busta S, Riedel A, Zollner G, Jost B. Evaluation of clinical trials by Ethics Committees in Germany: experience of applicants with the review of requests for opinion of the Ethics Committees - results of a survey among members of the German Association of Research-Based Pharmaceutical Companies (VFA). German Medical Science 2009;7:Doc07.

(150) Saginur R, Dent SF, Schwartz L, Heslegrave R, Stacey S, Manzo J. Ontario Cancer Research Ethics Board: lessons learned from developing a multicenter regional institutional review board. Journal of Clinical Oncology 2008 Mar 20;26(9):1479-82.

(151) Saito T. Ethics committees in Japanese medical schools. HEC Forum 1992;4(4):281-7.

(152) Sarpel U, Hopkins MA, More F, Yavner S, Pusic M, Nick MW, et al. Medical students as human subjects in educational research. Medical Education Online 2013;18:1-6.

(153) Schellings R, Kessels AG, Ter RG, Kleijnen J, Leffers P, Knottnerus JA, et al. Members of research ethics committees accepted a modification of the randomized consent design. Journal of Clinical Epidemiology 2005 Jun;58(6):589-94.

(154) Schick IC, Guo L. Ethics committees identify success factors: a national survey. HEC Forum 2001 Dec;13(4):344-60.

(155) Sengupta S, Lo B. The roles and experiences of nonaffiliated and non-scientist, members of institutional review boards. Academic Medicine 2003;78(2):212-8.

(156) Shah S, Whittle A, Wilfond B, Gensler G, Wendler D. How do institutional review boards apply the federal risk and benefit standards for pediatric research? Jama-Journal of the American Medical Association 2004;291(4):476-82.

(157) Sherwood ML, Buchinsky FJ, Quigley MR, Donfack J, Choi SS, Conley SF, et al. Unique challenges of obtaining regulatory approval for a multicenter protocol to study the genetics of RRP and suggested remedies. Otolaryngology-Head and Neck Surgery 2006;135(2):189-96.

(158) Silberman G, Kahn KL. Burdens on research imposed by institutional review boards: the state of the evidence and its implications for regulatory reform. [Review]. Milbank Quarterly 2011 Dec;89(4):599-627.

(159) Silverman H, Hull SC, Sugarman J. Variability among institutional review boards' decisions within the context of a multicenter trial. Critical Care Medicine 2001 Feb;29(2):235-41.

(160) Simek J, Zamykalova L, Mesanyova M. Ethics Committee or Community? examining the identity of Czech Ethics Committees in the period of transition. Journal of Medical Ethics 2010;36(9):548-52.

(161) Skrutkowski M, Weijer C, Shapiro S, Fuks A, Langleben A, Freedman B. Monitoring informed consent in an oncology study posing serious risk to subjects. IRB 1998 Nov;20(6):1-6.

(162) Sleem H, Abdelhai RA, Al-Abdallat I, Al-Naif M, Gabr HM, Kehil ET, et al. Development of an accessible self-assessment tool for research ethics committees in developing countries. Journal of Empirical Research on Human Research Ethics 1997 Aug;5(3):85-96.

(163) Sleem H, El-Kamary SS, Silverman HJ. Identifying structures, processes, resources and needs of research ethics committees in Egypt. BMC Medical Ethics 2010;11:12.

(164) Smith M, Doyle F, Mcgee HM, De La Harpe D. Ethical approval for national studies in Ireland: an illustration of current challenges. Irish Journal of Medical Science 2004;173(2):72-4.

(165) Speckman JL, Byrne MM, Gerson J, Getz K, Wangsmo G, Muse CT, et al. Determining the costs of institutional review boards. [References]. IRB: Ethics & Human Research 2007 Mar;(2):7-13.

(166) Stair TO, Reed CR, Radeos MS, Koski G, Camargo CA, MARC Investigators.Multicenter Airway Research Collaboration. Variation in institutional review board responses to a standard protocol for a multicenter clinical trial. Academic Emergency Medicine 2001 Jun;8(6):636-41.

(167) Stark A, Tyson J, Hibberd P. Variation among institutional review boards in evaluating the design of a multicenter randomized trial. Journal of Perinatology 2010;30(3):163-9.

(168) Sugarman J, Getz K, Speckman JL, Byrne MM, Gerson J, Emanuel EJ. The cost of institutional review boards in academic medical centers. New England Journal of Medicine 2005;352(17):1825-7.

(169) Sumathipala A, Siribaddana S, Hewage S, Lekamwattage M, Athukorale M, Siriwardhana C, et al. Informed consent in Sri Lanka: a survey among ethics committee members. BMC Medical Ethics 2008;9:10.

(170) Taylor HA, Currie P, Kass NE. A study to evaluate the effect of investigator attendance on the efficiency of IRB review. Irb 2008;30(1):1-5.

(171) Taylor HA, Chaisson L, Sugarman J. Enhancing communication among data monitoring committees and institutional review boards. Clinical Trials 2008;5(3):277-82.

(172) Thompson IE, French K, Melia KM, Boyd KM, Templeton AA, Potter B. Research Ethical Committees in Scotland. British Medical Journal 1981;282(6265):718-20.

(173) Thompson DA, Kass N, Holzmueller C, Marsteller JA, Martinez EA, Gurses AP, et al. Variation in local institutional review board evaluations of a multicenter patient safety study. Journal for Healthcare Quality 2012 Jul;34(4):33-9.

(174) Tsan MF, Smith K, Gao B. Assessing the quality of human research protection programs: The experience at the Department of Veterans Affairs. [References]. IRB: Ethics & Human Research 2010 Jul;(4):16-9.

(175) Tsan MF, Nguyen Y, Brooks R. Assessing the Quality of VA Human Research Protection Programs: VA vs. Affiliated University Institutional Review Board. Journal of Empirical Research on Human Research Ethics 2013 Apr;8(2):153-60.

(176) Tsan MF, Nguyen Y, Brooks R. Using quality indicators to assess human research protection programs at the Department of Veterans Affairs. [References]. IRB: Ethics & Human Research 2013 Jan;(1):10-4.

(177) Tully J, Ninis N, Booy R, Viner R. The new system of review by multicentre research ethics committees: prospective study. British Medical Journal 2000;320(7243):1179-82.

(178) Valdez-Martinez E, Trumbull B, Gardunno-Espinosa J, Porter JDH. Understanding the structure and practices of research ethics committees through research and audit: a study from Mexico. Health Policy 2005;74(1):56-68.

(179) Valdez-Martinez E, Turnbull B, Garduno-Espinosa J, Porter JD. Descriptive ethics: a qualitative study of local research ethics committees in Mexico. Developing World Bioethics 2006 May;6(2):95-105.

(180) Van Essen GL, Story DA, Poustie SJ, Griffiths MM, Marwood CL. Natural justice and human research ethics committees: an Australia-wide survey. Med J Aust 2004 Jan 19;180(2):63-6.

(181) van Luijn HE, Aaronson NK, Keus RB, Musschenga AW. The evaluation of the risks and benefits of phase II cancer clinical trials by institutional review board (IRB) members: a case study. Journal of Medical Ethics 2006 Mar;32(3):170-6.

(182) Vick CC, Finan KR, Kiefe C, Neumayer L, Hawn MT. Variation in Institutional Review processes for a multisite observational study. Am J Surg 2005 Nov;190(5):805-9.

(183) Vogeli C, Koski G, Campbell EG. Policies and management of conflicts of interest within medical research institutional review boards: results of a national study. Academic Medicine 2009 Apr;84(4):488-94.

(184) Vulcano DM. The development and acceptance of a simple tool to aid IRB compliance. Quality Management in Health Care 2012 Jul;21(3):203-8.

(185) Vulcano DM. Frustrations in benchmarking IRBs: reflections after analyzing the United States' IRB registration database. Journal of Empirical Research on Human Research Ethics 2012 Apr;7(2):34-6.

(186) Wagner TH, Bhandari A, Chadwick G, Nelson DK. The cost of operating institutional review boards (IRBs). Academic Medicine 2003;78(6):638-44.

(187) Wagner TH, Cruz AME, Chadwick GL. Economies of scale in institutional review boards. Medical Care 2004;42(8):817-23.

(188) Wagner TH, Murray C, Goldberg J, Adler JM, Abrams J. Costs and benefits of the national cancer institute central institutional review board. Journal of Clinical Oncology 2010 Feb 1;28(4):662-6.

(189) Watson LF, Rayner JA, Lumley JM. Hospital ethics approval for a population-based case-control study of very preterm birth. Australian Health Review 2007 Nov;31(4):514-22.

(190) Whitney SN, Alcser K, Schneider C, McCullough LB, McGuire AL, Volk RJ. Principal investigator views of the IRB system. International Journal of Medical Sciences 2008;5(2):68-72.

(191) Wichman A, Kalyan DN, Abbott LJ, Wesley R, Sandler AL. Protecting human subjects in the NIH's Intramural Research Program: a draft instrument to evaluate convened meetings of its IRBs. Irb: a Review of Human Subjects Research 2006 May;28(3):7-10.

(192) Williams-Jones B, Potvin MJ, Mathieu G, Smith E. Barriers to research on research ethics review and conflict of interest. IRB: Ethics & Human Research 2013 Sep;(5):14-9.

(193) Willison DJ, Emerson C, Szala-Meneok KV, Gibson E, Schwartz L, Weisbaum KM, et al. Access to medical records for research purposes: varying perceptions across research ethics boards. Journal of Medical Ethics 2008 Apr;34(4):308-14.

(194) Wisner KL, Conley RR, Taylor SF, Kosten T, Rapaport MH, Brown LS. Researcher experiences with IRBs: a survey of members of the American College of Neuropsychopharmacology. IRB 2011 Sep;33(5):14-20.

(195) Wu MH, Liao CH, Chiu WT, Lin CY, Yang CM. Can we accredit hospital ethics? A tentative proposal. Journal of Medical Ethics 2011 Aug;37(8):493-7.

(196) Yawn BP, Graham DG, Bertram SL, Kurland MJ, Dietrich AJ, Wollan PC, et al. Practice-based Research Network Studies and Institutional Review Boards: Two New Issues. Journal of the American Board of Family Medicine 2009;22(4):453-60.

(197) Zeeneldin AA. Adherence of non-pharmaceutically sponsored oncology trial protocols to the International Conference on Harmonization (ICH) guidelines in an academic institution outside the ICH jurisdictions and the impact of IRB implementation on this adherence. Journal of Egyptian National Cancer Institute 2013 Jun;25(2):71-8.

(198) Zhou P, Xue D, Wang T, Tang ZL, Zhang SK, Wang JP, et al. Survey on the function, structure and operation of hospital ethics committees in Shanghai. Journal of Medical Ethics 2009 Aug;35(8):512-6.
