# Supplementary material for: A Scoping Review of Empirical Research Relating to Quality and Effectiveness of Research Ethics Review
Source: PLoS One. 2015 Jul 30;10(7):e0133639. doi: 10.1371/journal.pone.0133639 (PMC4520456; doi:10.1371/journal.pone.0133639)
Supplement: S2 Table — (DOC) [file pone.0133639.s003.doc]

S2 Table : Identified measures or tools for evaluating research ethics review

| **Author** | **Reference** | **Name of Tool** | **Description** | **Validated?** |
| --- | --- | --- | --- | --- |
| Ateudjieu J et al | (14) | TRREE Needs for Africa | 3 module training course for members of African RECs created after needs assessment in three African countries. | No |
| Douglass AJ et al | (47) | Monitoring of Health Research | 1-2 hour interviews with Principal Investigators as part of an active monitoring program and to assess the effectiveness of such a program for future monitoring by ethics committees. | No |
| Feldman JA et al | (60) | Sample Survey Instrument | Pilot study to measure the perceptions of present IRB members about the performance of their own panel with regards to efficiency, procedures and outcomes. | No |
| Karunaratne AS et al | (91) | Research Participant/Researcher Questionnaire | Identify major areas of concern and assess the prevalence of the most salient of these by interviewing/questionnaires for researchers and research participants. | No |
| Keith-Spiegel P et al  Reeser JC et al | (93), (145) | IRB RAT | List of 45 characterizations of IRB functions and activities. Clustered into eight themes. Participants assessed the relative importance of these themes. Asked to spread the ratings across the full range of response categories. Asked both how each item was rated with regards to their own work and to an ideal REB | No |
| Sleem H et al | (163) | Research Ethics Committee (REC) Quality Assurance Self-Assessment Tool | Creation of a self-assessment tool that would measure the effectiveness in terms of protecting the rights and welfare of research participants. | No |
| Tsan MF | (174) | Quality Indicators for Assessing Human Research Protection Programs in the Department of Veterans Affairs | Quality indicators that emphasize assessing the outcome of the human research protection program  16 Quality indicators were approved:. | No |
| Vulcano DM | (184) | Placemat Tool | Federal criteria for the most common IRB decisions was placed on the placemats and distributed | No |
| Wichman A | (191) | Draft Evaluation Instrument for Use in Convened NIH IRB Meetings | Three parts: Regulatory Requirements, IRB Thoroughness, and meeting dynamics | No |
| Wu MH | (195) | Draft hospital ethics accreditation standards | Included 6 chapters, and 62 standards for hospital accreditation. Establishment of a research ethics included within this | No |
